# EF-Hand Binding Order and Conformational Control of Lanthanide Ion Binding in *M. extorquens* Lanmodulin

**DOI:** 10.1021/acs.biochem.6c00566

**Published:** 2026-10-13

**Authors:** Claire C. Mammoser, Natalie A. Savastio, Joseph A. Cotruvo

**Affiliations:** Department of Chemistry, The Pennsylvania State University, University Park, Pennsylvania 16802, United States

## Abstract

Biomolecular chelators have emerged recently as potential alternatives to conventional, energy-intensive methods for the challenging separation of rare earth elements (REEs), including the lanthanide (Ln^III^) ions. Lanmodulin (LanM) from *Methylobacterium extorquens* is the archetype for highly selective biological REE chelation and has been applied for recovery and separations of REEs. LanM’s high affinity and selectivity are driven by a large disorder-to-order conformational transition in response to cooperative binding of Ln^III^ ions to three of its EF-hand motifs, with one site being more labile than the other two. Definition of the order of Ln^III^ ion binding and the structural interactions contributing to cooperativity has been elusive but is essential for engineering of LanM and other REE-binding proteins. Here, we use a combination of mutagenesis and spectroscopic methods (UV absorbance, circular dichroism, luminescence, and NMR spectroscopies) to achieve both aims. We identify a salt bridge (K93-D56) between LanM’s two primary helices, disruption of which inactivates one of the metal-binding sites. Together, our results suggest, contrary to previous hypotheses, that EF1 and EF2 are the tightest sites, which are sufficient to organize most of the protein’s tertiary structure; EF3 is the labile, third binding site; and REEs, circumneutral pH, and the salt bridge are all essential for LanM’s full conformational change. These results help to define the conformational landscape underlying LanM’s selective REE recognition and provide design principles for engineering REE-binding proteins for recovery and separation applications.

## Graphical abstract

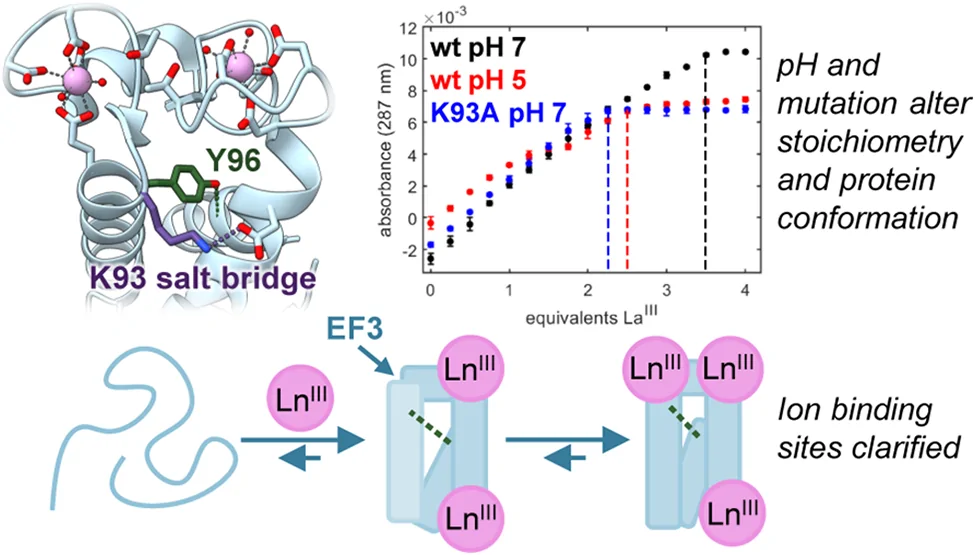

## Introduction

Increased awareness of the chemical and geopolitical challenges associated with the separation of rare earth elements (REEs), including the 15 lanthanides,^1–4^ has coincided with the discovery of essential biological roles for certain trivalent lanthanide (Ln^III^) ions.^5–8^ The promise of biological ligands to potentially achieve more efficient REE separations under milder conditions than conventional processing is perhaps most developed with lanmodulins (LanMs), a family of small proteins with high affinity and unprecedented selectivity for REEs over non-REEs.^9–12^ LanMs have been implemented in research- and larger-scale efforts for recovery and separations of REEs,^13–20^ a difficult feat due to REEs’ conserved +3 charge and similarity in ionic radius and coordination number preferences.^1,21^ The cooperative binding exhibited by LanM’s three REE-binding sites offers potential benefits for enhancing separations,^10,12^ but it also adds complexity in terms of implementation.^22^ Therefore, detailed elucidation of LanM’s REE binding would both shed light on its mechanisms of selective REE recognition and provide guidance as to how to capture the essence of the full protein’s selectivity (both for REEs over non-REEs and between REEs) in simpler peptide frameworks^23–28^ or even in synthetic materials.^29–32^ Here, we use a panel of spectroscopic methods to reassess and revise a model for the order of binding site occupancy in the archetypal LanM from *Methylobacterium extorquens* (*Mex*-LanM), revealing an unexpectedly crucial role of an interhelical salt bridge in the third binding event and in the protein’s full, REE-specific conformational change.

*Mex*-LanM, like all lanmodulin-family proteins,^11^ contains four EF-hand motifs, each comprising a 12-residue, carboxylate-rich metal-ion binding loop flanked by an “entering” α-helix N-terminal to the loop and an “exiting” α-helix C-terminal to the loop (Figure 1a,b). (In this work, “LanM” refers to *Mex*-LanM unless otherwise specified, and “EF-hand” denotes the metal-binding loop.) The vast majority of canonical EF-hand proteins bind Ca^II^ ions,^33^ but LanM instead binds REE^III^ ions in the first three of its EF-hands (EF1, EF2, and EF3; EF4 is much weaker and its metal ion binding can be disregarded in solution). LanM binds REE^III^ ions with picomolar apparent *K*_d_ values at pH 7, favoring the larger, light REEs, especially Nd^III^, but also including La^III^.^10,12^ Its selectivity against other ions with similar coordination preferences, including Ca^II^, is remarkable, > 10^8^-fold^10^ based on apparent *K*_d_ and ∼ 10^12^-fold based on competition assays.^34^ Binding of Ln^III^ ions (and Y^III^) induces major conformational changes from a state with little secondary and tertiary structure to an ordered conformation (Figure 1c), which can be observed spectroscopically by multiple methods (Figure 1a). This transition includes significant ordering of the protein’s secondary structure (α-helix formation, as observed using circular dichroism (CD) spectroscopy) as well as tertiary structural formation (including specific interhelical hydrogen bonding evident in the NMR and X-ray structures of Ln^III^-bound LanM).^12,35^ The binding-induced conformational change appears to be essential for LanM’s affinity and selectivity, as peptides derived from LanM’s individual EF-hands, and even a sequence comprising EF2, EF3, and the intervening 13 residues of *Mex-*LanM, have only micromolar affinity and poorly defined selectivity against non-REEs.^23,24^

**Figure 1:**
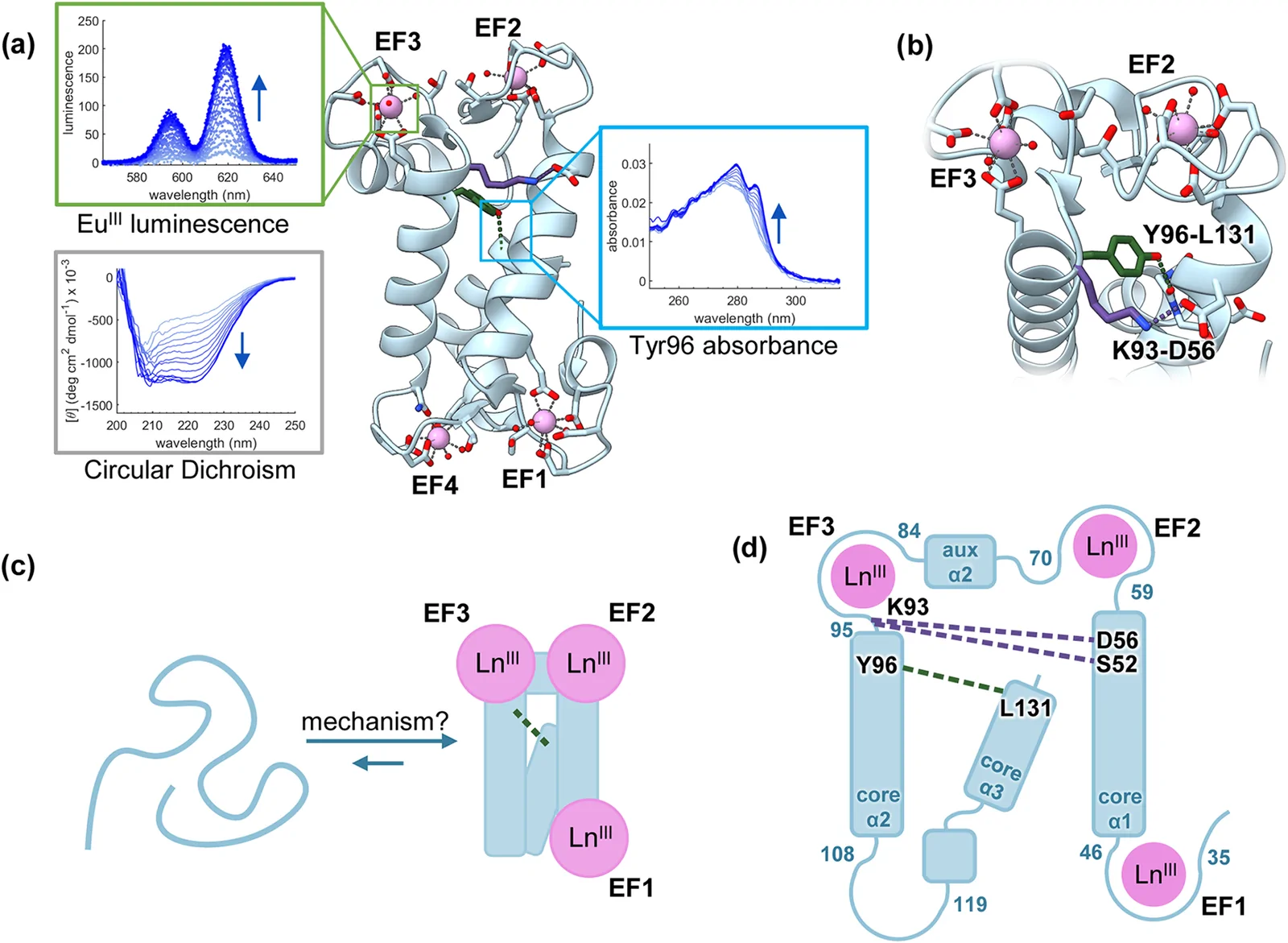
(a) X-ray crystal structure of Nd^III^-bound *Mex*-LanM (PDB: 8FNS) showing positions of Ln^III^ ions. Spectra show representative signal change from addition of Ln^III^ ions by three spectroscopic methods. Despite the presence of an Nd^III^ ion in EF4 in the protein as crystallized, EF4 is the weakest site, not occupied under our conditions, and unlikely to be occupied in vivo. (b) Key interhelical interactions and EF-hand structure in LanM; the positions of the K93-D56 salt bridge and Y96 hydrogen bond with the L131 backbone carbonyl are denoted. (c) General representation of Ln^III^ ion-induced conformational change in LanM. The dotted line denotes the Y96-L131 hydrogen bond. In the NMR structure with Y^III^, Y96′s hydrogen bond is formed with the adjacent carbonyl (N130). (d) Cartoon representation of LanM secondary structural topology and key interhelical interactions. Numbers in blue correspond to the residues at the start and end of each EF hand. The Nd^III^ ion occupying EF4 in the X-ray structure has been removed.

Based on initial studies using Hill analysis and later work using an Adair-type binding model, binding of the initial two Ln^III^ ions to LanM shows positive cooperativity and accompanies the major conformational change in LanM, accounting for two-thirds of the final α-helical formation observed by CD spectroscopy.^10,22^ A third Ln^III^ equivalent binds LanM slightly more weakly at pH 7 to form the full tertiary structure, but this third event is strongly pH-dependent and is weak at pH 5.^10,36^ By contrast, the tightest two sites maintain binding even below pH 3.0.^37^ Characterization of the folding intermediate before the third binding event would help to define (1) the tightest-binding EF-hand pair and the location of the third binding event, (2) what portions of secondary structural ordering accompany the third binding event, (3) when the tertiary structure is formed, and (4) why the third event is so pH-dependent. The cooperative folding of the protein has made these questions difficult to disentangle, but they are important for mechanism-guided engineering of minimal models of LanM. Previously, we have attempted to address this challenge by aiming to probe each EF-hand individually using sensitization of Tb^III^ luminescence by Trp residues incorporated into each site.^38^ Based on the results, we proposed that EF2 and EF3 were the highest-affinity cooperative EF-hands, a model which has been used to guide the design of a size-minimized LanM-derived peptide.^23,38^ However, the surprisingly weak binding of that peptide, coupled to the acknowledged structural perturbations and variables introduced by the Trp substitutions, led us to reexamine the four questions above using the wild-type (wt) protein.

Here, we define the tightest-binding EF-hands and the third binding site with greater certainty, and we reveal specific interactions in the protein’s structure required to maintain high affinity and cooperativity for Ln^III^ ions that provide insight into the conformation of the folding intermediate. We identified an outer-sphere substitution, K93A (Figure 1b,d), that surprisingly inactivates one of the metal-binding sites. Using a combination of spectroscopic methods (UV absorbance, CD, luminescence, and NMR spectroscopies) to probe this variant in comparison with wt LanM at pH 5 and pH 7, we present a revised model for conformational control of Ln^III^ stoichiometry which highlights (1) long-range tertiary structure interactions being formed upon the first two binding events, (2) EF3 as the third binding site, and (3) the specific requirements for REEs, circumneutral pH, and a salt bridge involving K93 and D56 for LanM’s full conformational change. These results fill in important details of the conformational landscape underlying LanM’s selective REE recognition, providing key mechanistic information and design principles not just for this protein but also for engineering of protein/peptide-based separation processes more generally.

## Experimental Section

Methods for the expression and purification of wt *Mex*-LanM and all LanM variants are described in the SI Methods. Details of spectroscopic data acquisition and interpretation (Eu^III^ luminescence spectroscopy, CD spectroscopy, UV absorbance spectroscopy, competition experiments with xylenol orange), as well as spectra contributing to the analysis, are also provided in the SI. NMR spectra were acquired using uniformly ^15^N-labeled wt or K93A *Mex*-LanM; details of expression of the labeled proteins and preparation of NMR samples, along with details of data processing and interpretation, can also be found in the SI.

## Results

### Approach to Study LanM’s Conformational Response

LanM possesses several convenient spectroscopic handles that can report on its conformational changes (Figure 1a). Because the Ln^III^ ion-induced conformational change involves the formation of α-helices from a disordered apoprotein, CD spectroscopy can be used to track the formation of LanM’s secondary structure; increased magnitude of the molar ellipticity at 222 nm, indicative of α-helix formation, has therefore been used as a proxy for Ln^III^ ion binding.^10^ Similarly, Ln^III^ binding-induced tertiary structural formation results in a hydrogen bond between Y96, the protein’s only intrinsic UV chromophore, and the protein’s C-terminal helix (Figure 1b,d). Growth of a sharp feature at 287 nm from Y96, which appears to report on hydrogen bonding^39–41^ with the backbone carbonyl of L131 can therefore be used to correlate formation of some degree of tertiary structure to Ln^III^ ion binding. Fluorescence emission of Y96 also decreases upon Ln^III^ ion binding, although it is less well understood whether this response is primarily sensitive to hydrogen bonding or to the environment (for example, the effective solvent dielectric) around the phenol ring. While each of these methods has been used to reveal important aspects of LanM’s Ln^III^ binding and conformational change, none is necessarily a direct measure of ion binding. Therefore, we have taken advantage of the luminescence of Ln^III^ ions such as Eu^III^ and Tb^III^, which can be excited directly to effect coordination-dependent changes in luminescence that can be interpreted as a direct readout of Ln^III^ coordination.^38,42^ The combination of luminescence spectroscopy, CD, and UV absorbance spectroscopies can thus probe Ln^III^ ion binding and the induced conformational changes in LanM.

### pH Dependence of Ln^III^ Stoichiometry Suggests Two Ions Mostly, But Not Fully, Order Tertiary Structure

Previous studies of *Mex*-LanM have demonstrated that, while binding of the first two Ln^III^ ions to LanM is not lost until pH < 3, binding of the third Ln^III^ ion is destabilized at pH values substantially closer to neutral,^36^ which can be observed at and below pH 5 when the free metal ion concentration is buffered using a chelator. We performed titrations using direct Eu^III^ luminescence, CD, and UV absorbance spectroscopies (Figure 2, additional spectra Figure S1) in order to evaluate the effects of pH on secondary and tertiary structure of the protein, with an initial focus on distinguishing this third binding event from the first two.

**Figure 2:**
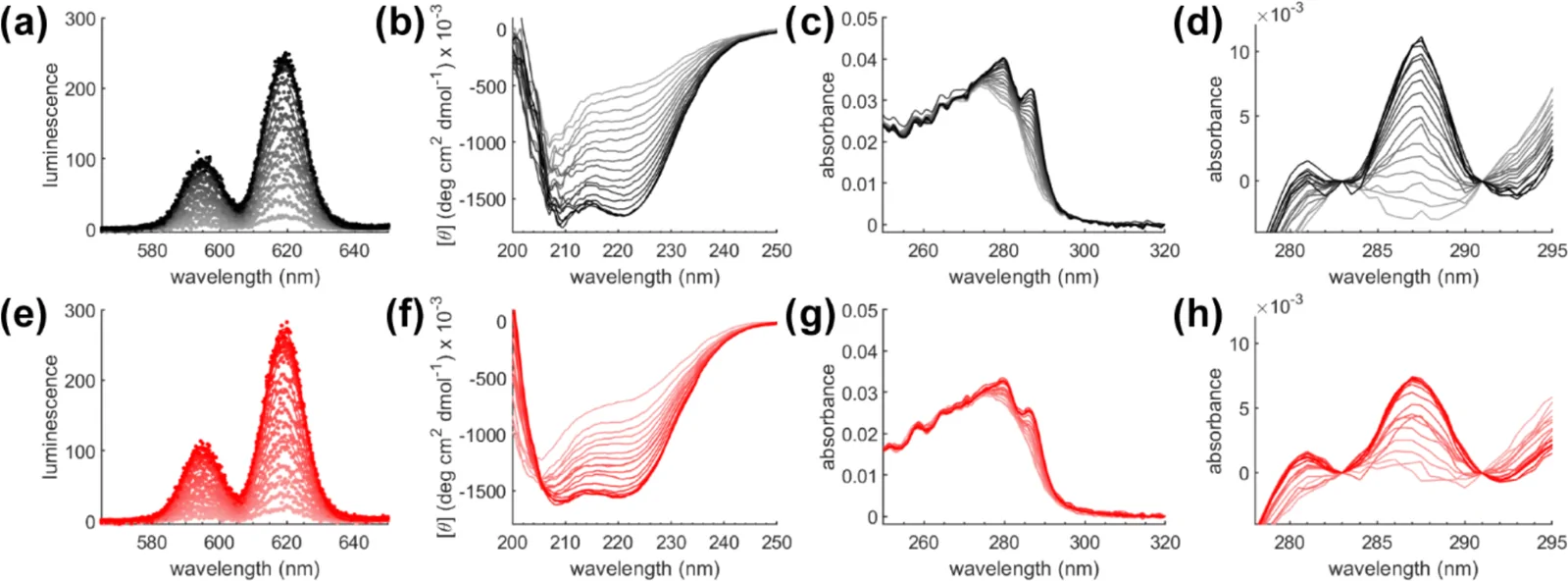
Spectra from representative titrations of 20 μM LanM with Eu^III^ (a, e) or La^III^ (b–d, f–h) (0.25 equiv per titration point) at pH 7.0 (a–d) and pH 5.0 (e–h). (a, e) Direct Eu^III^ luminescence spectra (λ_ex_ = 394 nm) from representative titrations. (b, f) CD spectra from representative titrations. (c, g) UV absorbance spectra from representative titrations. (d, h) Corrected spectra from panels (c) and (g); a linear baseline drawn between 283 and 291 nm was used to isolate the absorbance resulting from hydrogen bonding to Y96 and reduce spectrum-to-spectrum variability within a titration.

Direct excitation of Eu^III^ at 394 nm yields luminescence emission at ∼595 and ∼619 nm. The ^5^D_0_ → ^7^F_2_ emission feature around 619 nm is particularly sensitive to the coordination environment of the ion, while the emission peak at 595 nm is minimally affected.^43^ This makes Eu^III^ luminescence an effective means by which to quantify and characterize Eu^III^ coordination to a ligand (e.g., LanM) vs the aquated ion. At pH 7, as Eu^III^ is added to a solution of LanM, the luminescence at 619 nm increases linearly at a rate of ∼8 relative fluorescence units (rfu) per μM Eu^III^ under our experimental conditions (Figures 2a,e and 3a). After the titration reaches an end point at 3 equiv, the increase in luminescence at 619 nm decreases to ∼1 rfu/μM Eu^III^, reflective only of the additional Eu^III^ present in the solution. At pH 5, the slope of the increase in luminescence at 619 nm and the end point at 3 equiv is the same, indicating that the coordination of the Eu^III^ ions is similar at pH 5 and 7. The concentration of LanM used in this study is sufficiently high (20 μM) that binding of the third Ln^III^ ion cannot be separated from the two higher-affinity ions at pH 5 by this method (Figure 3a).

**Figure 3:**
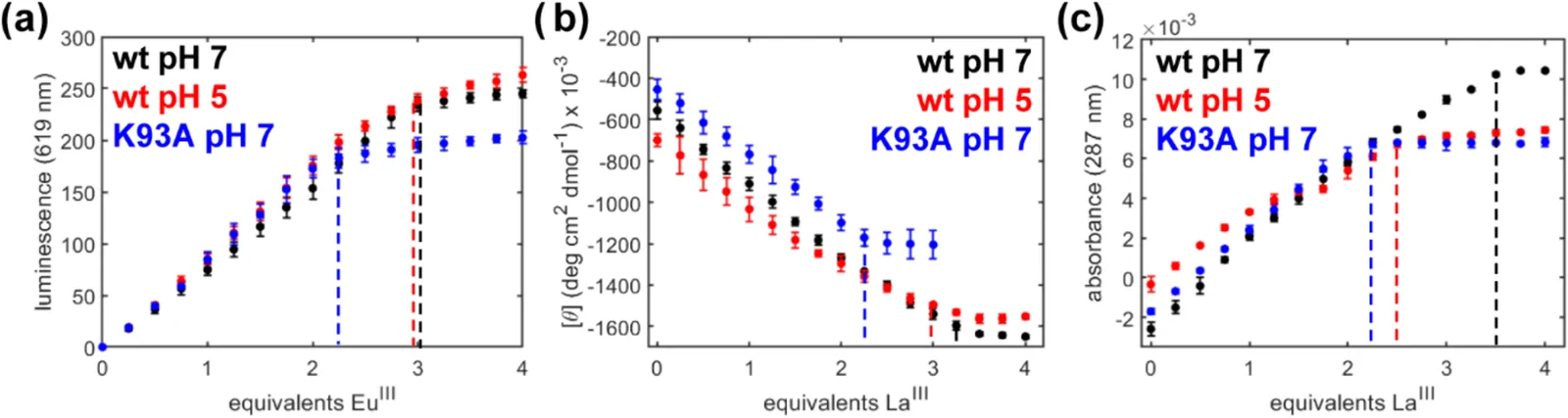
Spectroscopic response to binding of Ln^III^ ions to wt and K93A-LanM. (a) Luminescence spectroscopy (Eu^III^), (b) CD spectroscopy (La^III^), (c) UV absorbance spectroscopy of Y96 (La^III^). Values reported result from the subtraction of a linear baseline calculated between 283 and 291 nm, isolating the absorbance related to hydrogen bonding of Y96 from the underlying tyrosine absorbance. Protein concentrations are 20 μM for all experiments. Vertical dashed lines show the stoichiometry at which LanM is saturated with Ln^III^ ions, as indicated by a sharp decrease in slope of the response upon addition of Eu^III^ or La^III^. Error bars indicate the standard deviation from measurements of three separate samples.

We next performed similar stoichiometric titrations with La^III^ using circular dichroism (CD) spectroscopy. La^III^ was selected both as a representative light Ln^III^ ion and because it is diamagnetic, facilitating straightforward interpretation of NMR spectra below. Based on chelator-buffered titrations, we have previously reported that approximately two-thirds of the overall α-helical formation in LanM is associated with binding of the first two Ln^III^ ions, while binding of the third Ln^III^ ion is associated with the remaining third.^10,36^ As in titrations using Eu^III^ luminescence, LanM concentrations of 20 μM favor binding of the third La^III^ ion such that it cannot be observed as a separate, secondary binding event in stoichiometric titrations. The final molar ellipticity at 222 nm, a wavelength associated with α-helix formation, was similar at pH 7 and pH 5 (−1,600,000 deg cm^2^ dmol^–1^) (Figures 2b,f and 3b), indicating that the extent of secondary structural ordering is the same at each pH value. However, the magnitude of the initial molar ellipticity value is slightly greater at pH 5 than at pH 7, indicating that at pH 5, the protein may be partially preordered prior to binding of Ln^III^ ions. A potential mechanism for this effect is that the higher proton concentration in solution neutralizes some charge in the highly negatively charged EF hands, allowing them to take on a more holoprotein-like conformation, which supports minor α-helix formation.

Whereas spectral responses from Eu^III^ luminescence (probing ion coordination) and from CD spectroscopy (probing LanM’s secondary structural transition) were broadly similar at each pH studied, the absorbance of Y96, the residue immediately following EF3, in response to La^III^, suggests differences in tertiary structure. In the Ln^III^-bound holoprotein, the Y96 phenolic OH forms an interhelical hydrogen bond with the backbone CO of L131 in the C-terminal helix. On the basis of other systems, the growth of two sharp absorbance features at 280 and 287 nm as Ln^III^ ions are titrated into the protein is interpreted as reporting on this hydrogen-bonding interaction (Figures 1b and 2c,g).^39^ Thus, these features can be used as a reporter of tertiary protein folding (Figure 1d). In line with the CD results (Figure 3b), at pH 5, the starting absorbance at 287 nm is higher than that at pH 7, suggesting that the environment occupied by Y96 in the apoprotein is altered by the lower pH to be slightly more holoprotein-like (Figure 3c).

More importantly, the overall absorbance change from Y96 at 287 nm is nearly twice as large at pH 7 as at pH 5. At pH 7, absorbance at 287 nm linearly increases by 0.012 absorbance units (AU) over three equivalents of added La^III^, while at pH 5, the magnitude of the increase is only ∼0.07 AU (Figures 2c,d,g,h and 3c), and it only occurs over the first two La^III^ binding equivalents. While the slope of the ΔA_287 nm_ plot is similar for the first binding equivalent under both conditions, it is noticeably lower for the second binding equivalent at pH 5. The decreased holoprotein absorbance at pH 5 indicates that the final environment of the Y96, and therefore the surrounding protein conformation, differs from that at pH 7 and is unaffected by the binding of the third metal ion. While this observation could arise from differences in electrostatics around the residue, such effects generally do not approach the magnitude observed here (∼1/3 of the total change).^39^ For this reason, and since the specific signal observed at 287 nm is known to arise from hydrogen bonding of Tyr phenols,^39^ we postulate that the decrease in maximum absorbance intensity observed at pH 5 results from a weakened Y96-L131 hydrogen bond. This interpretation implies that lower pH values induce a deviation in LanM’s Ln^III^-bound tertiary structure that makes communication between Y96 and the C-terminus, and perhaps further tertiary structure change, insensitive to the third binding event, even though that Ln^III^ ion still binds (at least at pH 5).

Together, while Eu^III^ luminescence and CD indicate that three equivalents of Ln^III^ ions associate with LanM at both pH 5 and 7 under these conditions, the Y96 absorption data indicate that only the tightest two La^III^ equivalents contribute to the protein’s (incomplete) tertiary structural change at pH 5. At the same time, at both pH 5 and 7, the CD data indicate that 2 binding equivalents are not sufficient to fully order the secondary structure of the protein, suggesting that the third binding equivalent is responsible for some helix formation but perhaps is of lesser importance for ordering of the tertiary structure (i.e., the packing of the protein’s central helices). In other words, secondary and tertiary structure formation is partially decoupled: at pH 5, full secondary structure forms but the full tertiary structure does not, whereas at pH 7, much of the tertiary structure may form even before the third binding event orders the final helix (*vide infra*), in line with the previously observed higher affinity of the third event under these conditions.

### Mutation of a Central Salt Bridge Defines the Importance of Interhelical Cooperativity

The implication of Y96, the residue immediately following EF3, and its vicinity being perturbed at pH 5 drew our attention to the presence of an interhelix hydrogen bond between the side chains of K93 and D56. K93 is the tenth residue in EF3 and the first residue in the exiting helix (core helix α2), and D56 is a residue on the adjoining core helix α1 and one turn before the first residue of EF2 (D59) (Figure 1d). K93 is the only residue of core helix α2 capable of forming an interhelical hydrogen bond between these helices, primarily with D56 and potentially with S52, the side chain of which is modeled in two conformations, one of which is in hydrogen-bonding distance of K93.^12^ We introduced a substitution, K93A, hypothesizing that removing K93′s hydrogen bond with D56 and/or S52 would disrupt communication between these helices (Figure 1b,d). Unexpectedly, this substitution abrogated binding of the third Ln^III^ ion: all methods of analysis (direct Eu^III^ luminescence, CD spectroscopy, UV absorbance of Y96) indicate that K93A-LanM binds only two equivalents of Ln^III^ ions at pH 7 (Figure 3, results at pH 5 in Figure S2). In each case, the decreased Ln^III^ binding stoichiometry was accompanied by a smaller difference in signal between apo- and holo-LanM than observed for the wt protein (at pH 7). Characterization of a D56A-LanM variant yielded very similar results to the K93A variant (Figures S3 and S4), confirming that removal of the K93-D56 salt bridge was the source of the decreased stoichiometry and signal change relative to wt LanM.

Eu^III^ luminescence spectroscopy of K93A-LanM shows a signal increasing linearly with approximately the same slope as in wt LanM, but the signal stops increasing around two equivalents of Eu^III^ per LanM. This result provides a direct indication that the K93A substitution decreases binding stoichiometry even at pH 7 rather than merely altering the effect of Ln^III^ binding on protein conformation (as in the effect of pH on the wt protein) (Figure 3a).

The final α-helix content observed by CD spectroscopy of K93A-LanM is approximately 2/3 that of wt LanM under the same conditions (Figure 3b), notably similar to the extent of helical content assigned to the first two binding events in wt protein.^10,36^ The Y96-reported tertiary fold accessed by the K93A-LanM holoprotein is also similar to that observed in wt LanM at pH 5 (approximately 2/3 of wt signal at pH 7) (Figure 3c). Subtle differences between Y96 signals of wt LanM at pH 5 and K93A-LanM at pH 7 can be observed; for example, the absorbance of Y96 in K93A-LanM increases linearly with La^III^ to two equivalents, while wt LanM displays more complex binding behavior at pH 5. However, the similarity of the absorbance of Y96 in spectra of K93A-LanM and D56A-LanM at pH 7 and wt LanM at pH 5 suggests that the tertiary conformation of the holoproteins is similar under these conditions.

The ablation of the third binding event with the K93A substitution led us to hypothesize that the apparent dissociation constants (*K*_d_) of the first two Ln^III^ binding events might also be weakened. Because the Y96 UV absorbance reports on tertiary structure, we used this method to measure apparent *K*_d_s for the La^III^–LanM complexes. While the apparent *K*_d_ values of the K93A variant are slightly (∼3-fold) weaker at pH 7 and at pH 5, the affinity of the D56A variant is essentially unaltered at pH 5 (Table 1 and Figure S5). These results suggest that there is not a major perturbation to binding of the first two ions in either variant, and since K93A- and D56A-LanMs each bind only two equivalents of Ln^III^ ions regardless of pH (within the range investigated), these results confirm that the conformational change associated with binding of those two equivalents is sufficient to drive the protein’s tight affinity. We therefore propose that the K93A variant can serve as a model for the limiting case of disfavored third-equivalent cooperativity in LanM that is approached in the wt protein at pH 5.

**Table 1:** Apparent Dissociation Constants (*K*_d_) and Hill Coefficients (*n*) for La^III^ to LanM, Measured by UV–Visible (Y96) Absorbance^a^

	pH 7		pH 5	
	*K*_d_ (pM)	Hill coefficient	*K*_d_ (pM)	Hill coefficient
wt	2.4 ± 0.4	3.2 ± 0.4	16 ± 3	1.7 ± 0.2
K93A	7.4 ± 1.5	2.2 ± 0.5	39 ± 9	1.7 ± 0.1
D56A	ND	ND	17 ± 3	2.0 ± 0.8
ΔEF1	ND	ND	46 ± 22	1.4 ± 0.7
ΔEF2	ND	ND	29 ± 8	1.4 ± 0.4
ΔEF3	ND	ND	65 ± 8	1.2 ± 0.4

a Binding of the third La^III^ ion is not resolved as a separate binding event at lower affinity at pH 5 for wt LanM, as reported by CD.^36^ ΔEF1, ΔEF2, and ΔEF3 refer to D_3,5,9_N substitution at the given EF hand. ND: not determined.

To summarize the initial insights from the removal of the K93-D56 salt bridge, altering this single interaction between the core helices of LanM is sufficient to abrogate the binding of the third Ln^III^ equivalent. In many ways, the effects of this substitution on the tertiary structure of LanM mimic those of the acidic (pH 5) condition, although it pushes those conformational effects to the extreme; the data suggest that K93A- and D56A-LanMs are unable to access a conformation that supports high-affinity third-equivalent binding. As K93 is the tenth residue in EF3, we suggest that the simplest explanation for the above data is that low pH and the K93A and D56A substitutions disrupt EF3, disfavoring binding at that site. The K93-D56 salt bridge and conformation of EF3 are almost certainly intimately connected to the positioning of Y96; at low pH or in the absence of the salt bridge, decreased UV absorbance from the Y96 hydrogen bond indicates that Y96 does not occupy the strongest hydrogen-bonding conformation, correlating with decreased affinity for Ln^III^ ions at EF3. Therefore, EF3 is the most likely binding site of the third Ln^III^ equivalent, while EF1 and EF2 comprise the tight-binding cooperative EF-hand pair.

### Binding of Ca^II^ Induces Altered Folding

In addition to low pH and amino acid substitutions, binding of non-REEs represents another potential perturbation of LanM structure. Ca^II^ has previously been shown to bind LanM with relatively low affinity and is associated with a lower magnitude of secondary structural ordering (as observed by CD spectroscopy) compared to that of Ln^III^ ions.^10^ We further investigated the impact of nonpreferred ion binding at pH 7 on the secondary and tertiary conformations of LanM by CD and UV absorbance, respectively. The CD spectroscopic signal of Ca^II^–LanM was less than that of Ln^III^-bound wt LanM under the same conditions and aligns with the La^III^-bound K93A variant (Figures 4a and S6a). Similarly, the absorbance of Y96 in the Ca^II^ holoprotein is approximately the same as that observed with La^III^ in the K93A variant and in wt LanM at pH 5 (Figures 4b and S6b,c). Because Ca^II^ binds relatively weakly to LanM, we cannot directly determine the binding stoichiometry. While a range of Ln^III^ ions induce the same change in Y96 absorbance as La^III^ does at pH 7 (Figure S7), the observation of a common conformational response to ion binding at slightly acidic pH, with amino acid substitution, or upon association of a nonpreferred ion argues that there may be a highly conserved suboptimal secondary and tertiary structure which is accessible to the protein under a wide range of perturbative conditions.

**Figure 4:**
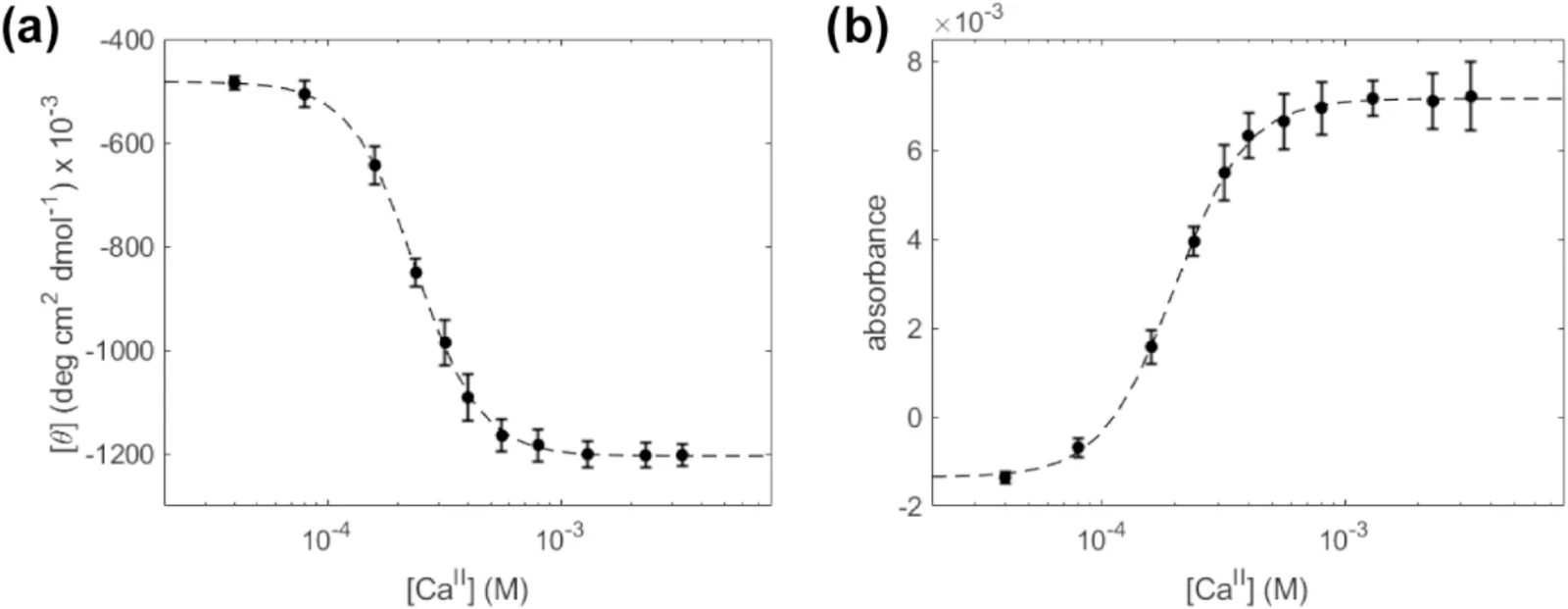
Binding of Ca^II^ ions to wt LanM (20 μM) at pH 7.0, monitored by (a) CD spectroscopy (*K*_d_ = 239 ± 9 μM, *n* = 3.1 ± 0.2) and (b) Y96 absorbance (*K*_d_ = 199 ± 2 μM, *n* = 2.9 ± 0.1). Values reported in panel (b) result from the subtraction of a linear baseline calculated between 283 and 291 nm, isolating the absorbance related to hydrogen bonding of Y96. Data were fitted to the Hill equation.

### NMR Spectroscopy Helps to Determine Structural Drivers of Decreased Cooperativity

The above results demonstrate that acidic pHs, the K93A or D56A substitution, and Ca^II^ impact the ability of LanM to access a conformation that supports cooperative tight binding of the third Ln^III^-binding equivalent. To further investigate the structural basis of this effect, we collected ^1^H–^15^N heteronuclear single quantum coherence (HSQC) NMR spectra of wt and K93A-LanM bound to La^III^ and Y^III^. Use of the K93A variant allowed us to access a state in which two equivalents of La^III^ or Y^III^ were bound per LanM at pH 7, a state which is inaccessible in the wt protein because the affinity of the third site is still high and linked cooperatively to binding at the first two sites, such that the doubly REE-bound state does not accumulate at pH 7.^35^ Therefore, our initial hypothesis was that comparison of K93A-LanM to the wt would reveal high chemical shift perturbation of the resonances assigned to either EF1, EF2, or EF3, facilitating identification of the site of the third binding event.

Resonances for Y^III^-bound wt LanM at pH 7 had been previously assigned as part of a structural study.^35^ Because those data were collected using a C-terminally His_6_-tagged LanM, we first collected an analogous untagged Y^III^-bound sample and used the previously determined resonance assignments to assign the spectrum of the untagged protein. As anticipated, some of these resonances showed small deviations from the prior data collected on tagged protein (and with La^III^ vs Y^III^) (Figures S8 and S9). Assignments of resonances in other spectra (La^III^-bound, variants) to specific residues were then determined based on the new Y^III^-bound wt spectrum (Figures S9–S13). Notably, the NMR structure of Y^III^-bound LanM does not explicitly show the K93-D56 salt bridge, and hydrogen bonding with the Y96 side chain is with the backbone CO of N130, rather than that of L131, as shown in the X-ray structure, which might reflect the His_6_ tag or a difference between Y^III^- and La^III^-bound proteins.^12,35^

A comparison of spectra of wt (3 equiv La^III^) and K93A-LanM (2 equiv La^III^) at pH 7 reveals that most resonances overlap well, indicating minimal to no change in the environment of those residues; however, certain resonances assigned to residues around the interface of the two core helices do shift, indicating perturbation at those sites (Figures 5a and S14a). While the localization of the affected residues may not be surprising at first glance due to their proximity to the mutated residue (K93) and its partner in the affected salt bridge (D56), it is notable that none of LanM’s four EF hands contain residues that are highly affected by the mutation. Contrary to our hypothesis that K93A-LanM would have a single, highly perturbed EF-hand when compared to wt LanM, only minor perturbation is observed at EF2 and EF3. EF1, EF4, and the C-terminal helix show the least perturbation, and remarkably little change from wt. This suggests that EF1 is one of the two tightest-binding sites. However, the NMR data alone do not allow us to determine whether it is EF2 or EF3 that is part of the initial cooperative pair. This determination is obscured by the low magnitude of the chemical shift perturbation observed even in EF2 and EF3, which indicates that both EF hands are largely preset in their final “holo” conformation even though it appears that only one of these sites is occupied by a La^III^ ion (the other La^III^ ion is presumably bound to EF1).

**Figure 5:**
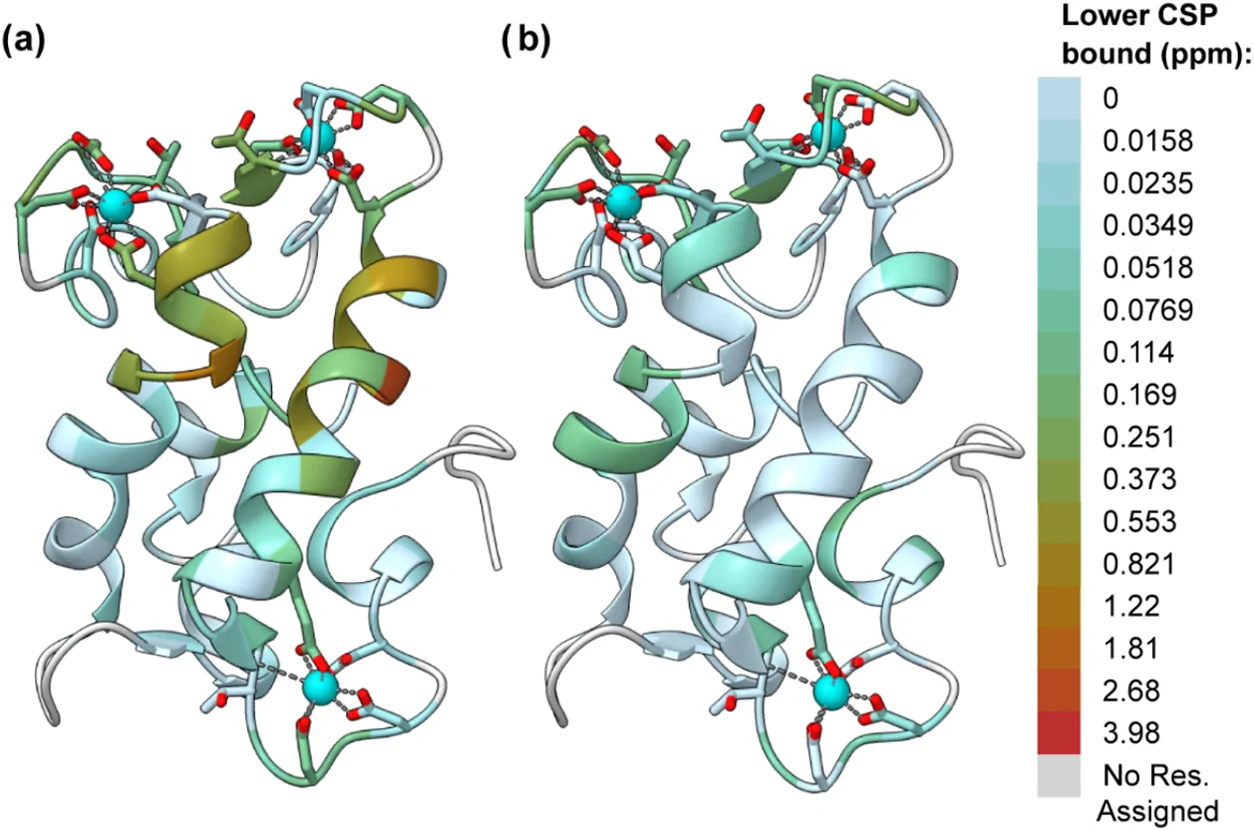
Structural representation (PDB: 6MI5) of the locations of residues whose resonances display chemical shift perturbation (CSP). CSP was determined from ^1^H–^15^N HSQC spectra of (a) wt LanM at pH 7 (3 equiv La^III^) vs K93A-LanM at pH 7 (2 equiv La^III^), and (b) wt LanM at pH 7 (3 equiv La^III^) vs wt LanM at pH 5 (2 equiv La^III^).

An analogous comparison of spectra of wt LanM at pH 7 (3 equiv La^III^) and at pH 5 (2 equiv La^III^) yields similar results (Figures 5b and S14b). The primary residues corresponding to resonances with the most chemical shift perturbation are at the interface of the core helices, again indicating that the third EF hand is preset for binding even with only two equivalents of La^III^ present. The most perturbed region is core helix α2 following EF3, but the overall magnitude of the chemical shift perturbation is smaller between wt LanM at pH 7 and pH 5 than when comparing with the K93A variant. It is notable, however, that the residues corresponding to the greatest spectral perturbation are localized similarly in both cases, even when no mutation was directly introduced. While these results could reflect that at pH 5, wt LanM experiences fast exchange between the states observed for wt- and K93A-LanM at pH 7, the distinct responses observed by other spectroscopic methods argue against this interpretation. Taken together, these observations support our conclusion from titration data that the K93A variant effectively represents an extreme case of the disfavored third-site cooperativity observed for the wt protein at pH 5.

### Ln^III^ Binding to LanM Variants with Disrupted EF Hands

The above results indicate that both low pH and disruption of interhelical contacts disfavor binding of the third binding equivalent to LanM, and they suggest EF1 as one of the first two sites, while they tentatively suggest EF3 might be the site of the third binding event. To further interrogate this matter, we designed three variants of LanM, each of which substitutes the Asp residues at positions 3, 5, and 9 in either EF1, EF2, or EF3 with Asn (D_3,5,9_N), denoted ΔEF1, ΔEF2, and ΔEF3. We anticipated that these substitutions would disfavor binding of Ln^III^ ions in the mutated EF hand while maintaining the overall structure of the protein as closely as possible, although neutralization of charge in a given EF hand might allow it to preorder in a manner not favored in the wt protein (*vide infra*). All of the variants were purified in good yield. The ΔEF1 variant showed some instability in solution over periods longer than 1–2 days, partially losing its conformational responsiveness (Figure S15), whereas minimal change was observed in wt LanM or any other variant over periods of approximately 1 week (see SI Methods). This observation anecdotally supports EF1′s criticality.

We first sought to verify that the variants were functional in Ln^III^ ion binding. Competition with xylenol orange (XO), a Ln^III^ ion-sensitive dye, has been used to estimate metal-binding stoichiometry of LanMs^10,44^ and other Ln^III^-binding proteins.^45^ With an estimated affinity (*K*_d_) at pH 6 in the low micromolar regime,^46^ XO should be intermediate to the tight (pM) binding sites in LanM and any binding sites weakened by pH or mutation (e.g., apparent *K*_d_ for the weakest Nd^3+^ binding site of wt LanM at pH 5 has been reported as ∼4 nM).^36^ Competitive titration (Figure 6a) of wt LanM and LanM variants with weakened EF hands revealed that the wt and ΔEF2 and ΔEF3 variants outcompeted XO for La^III^ up to ∼2 equiv, but the ΔEF1 variant was less competitive, binding ∼1.6 equiv La^III^. This result provided further evidence that EF1 is one of the tightest binding sites in LanM.

**Figure 6:**
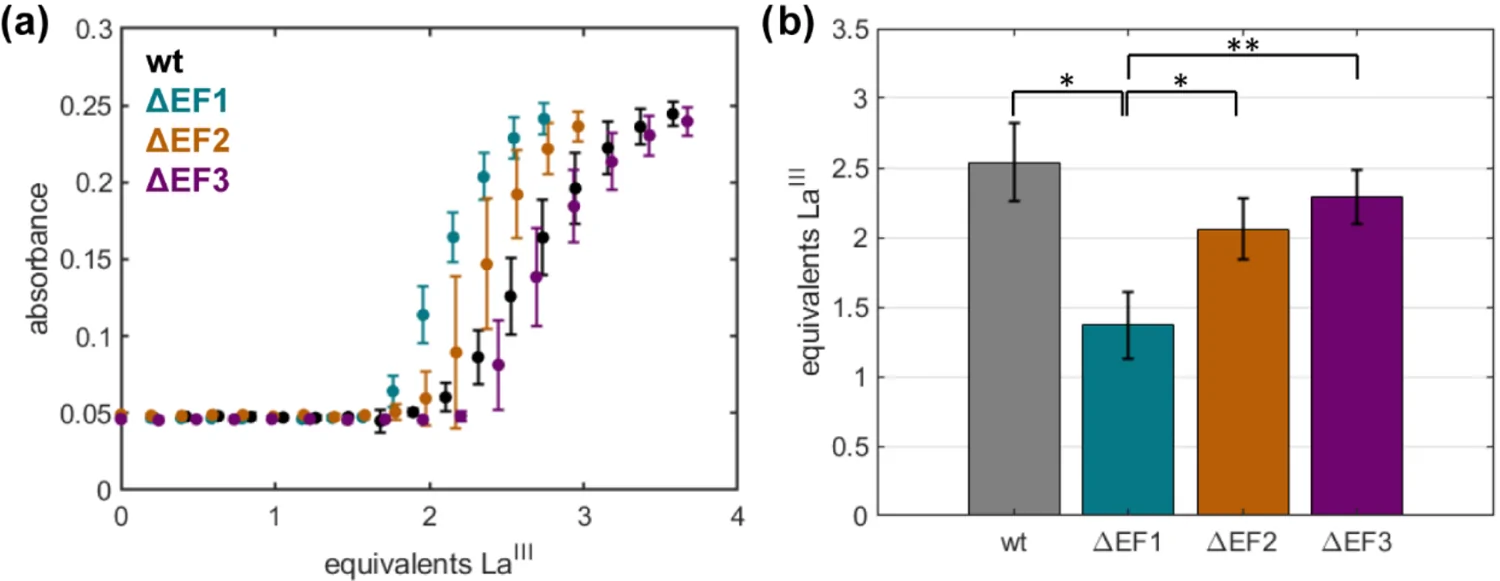
(a) Competition of wt and D_3,5,9_N variant LanMs (ΔEF1, ΔEF2, and ΔEF3, 10 μM) with XO (5 μM) at pH 6.0, during titration with La^III^ ions. The absorbance of XO at 575 nm increases when XO binds La^III^, and the point at which the absorbance increases beyond the starting value is taken as the stoichiometry of protein metal-binding sites that are tighter than XO. (b) Quantification of tightly bound La^III^ following passage through a spin desalting column (pH 5) from ICP-MS. Protein concentrations were determined by Pierce BCA assay. Error bars represent a standard deviation of triplicate samples; significance determined by a two-tailed *t* test is indicated by asterisks (*p < 0.05, **p < 0.01). See also Table S1.

Because the XO method is dependent on the relationship between the affinity of Ln^III^ ion for each site in LanM and for XO, the latter of which is not well-defined,^44^ and because it can only be used at pH 6 based on the properties of the indicator, we sought an additional method by which to clarify our findings. We incubated wt LanM and the variants with D_3,5,9_N substitutions with four equivalents of La^III^ and employed spin desalting columns to remove any loosely bound La^III^. This method is based on the kinetic lability of the ions from LanM rather than the affinity; while the two are related, “tight” binding in this case refers to a low probability of an ion dissociating and rebinding to LanM. We then analyzed the resulting solutions using ICP-MS to determine the concentration of La^III^ tightly bound to each protein sample. As in the competitive titrations with XO, at pH 5, wt LanM and the ΔEF2 and ΔEF3 variants bound La^III^ with similar tight-binding stoichiometries (2.0–2.5 equiv, Figure 6b). Meanwhile, the ΔEF1 variant bound only ∼1.4 equiv, again indicating that EF1 is essential for maintenance of the protein’s full Ln^III^ binding behavior. At pH 7, tight-binding stoichiometries are essentially unaltered for each ΔEF variant, and slightly increased in wt LanM, indicating that the wt protein’s third binding site may be more kinetically stable under these conditions and supporting a model which includes population of a more stable conformation by wt LanM at neutral pH (Table S1). K93A- and D56A-LanMs exhibit a loss of one binding equivalent at pH 7 relative to wt, as expected (Table S1).

The apparent *K*_d_s (pH 5) of each ΔEF variant are minimally affected by the mutation, showing only a small increase in apparent *K*_d_ (decrease in affinity), similar to observations with K93A-LanM (Table 1 and Figure S16). This result is especially notable because at pH 5, we anticipate that in two of the three variants, one member of the pair of core tight-binding EF hands should have disfavored binding due to the substitutions. It therefore seems likely that binding of only one ion is sufficient to template the protein for binding of the second ion, in line with the positive cooperativity expected for the binding of the first two Ln^III^ equivalents.^10,22^

### Occupancy of LanM’s EF Hands and Tertiary Structure

The experiments above, while pointing to the importance of EF1, were again not able to clearly discriminate between EF2 and EF3, motivating further characterization of the ΔEF variants. Direct Eu^III^ luminescence titrations revealed similar behavior for each variant as in the wt protein at both pH 7 and pH 5 (Figures 7a,d, S17a, and S18a). This result is likely for the same reason as the similarity of stoichiometric luminescence titrations of wt protein at pH 7 and pH 5: the relatively high concentrations (20 μM) of protein push the equilibrium toward binding, even at relatively disfavored EF hands. The final luminescence intensity differed among certain variants, particularly ΔEF3, perhaps reflecting differences in the coordination environment (especially hydration) of one or more of the Eu^III^ ions. At pH 5, the equivalence point of the ΔEF2 titration also appeared to be earlier, toward 2 equiv, than the other proteins. Nevertheless, the results were broadly similar and indicated that the substitutions did not fully ablate Eu^III^ ion binding at any site.

**Figure 7:**
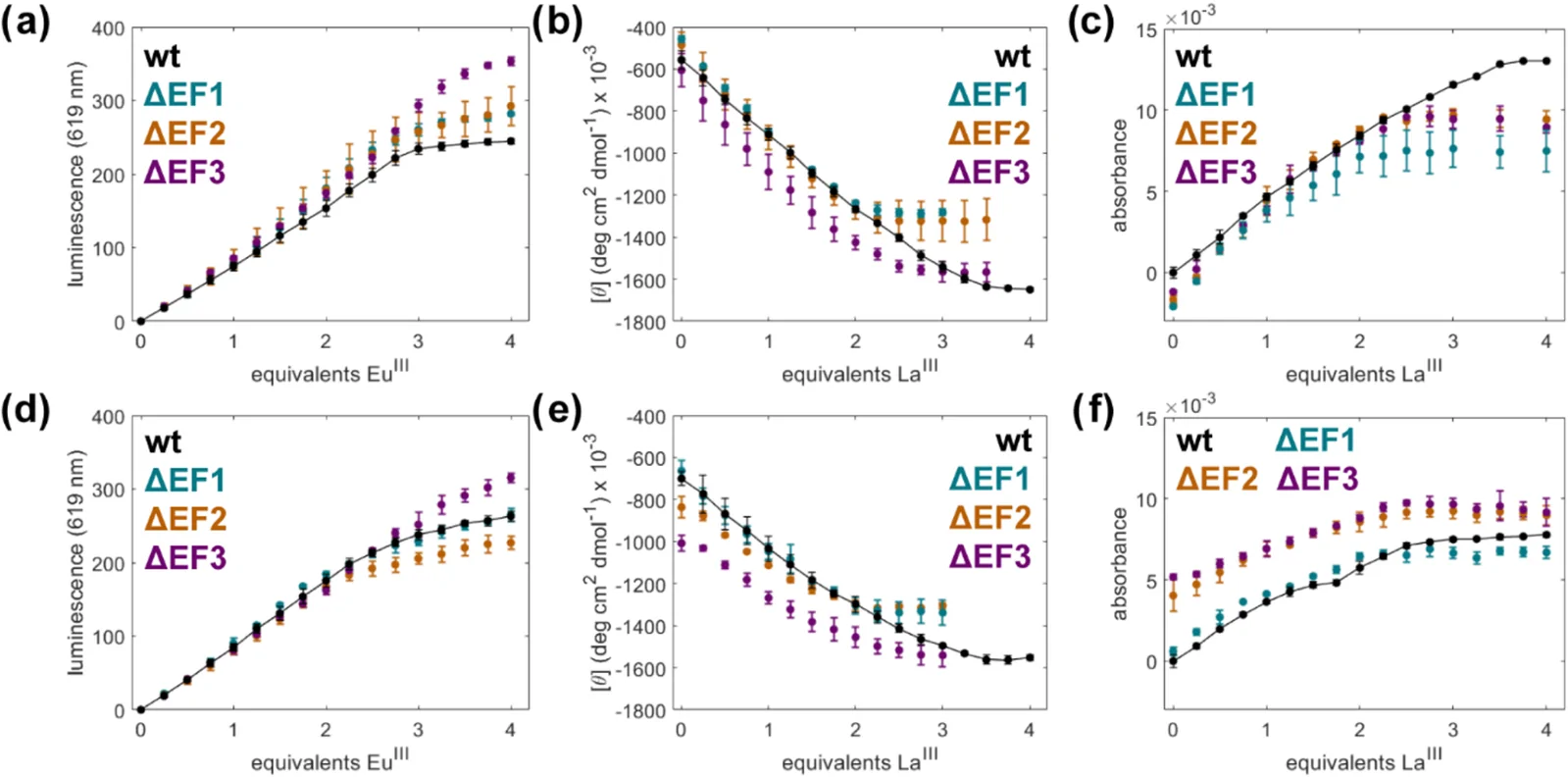
Spectroscopic response of wt LanM and ΔEF1, ΔEF2, and ΔEF3 variants to binding of Ln^III^ ions at pH 7 (a–c) and pH 5 (d–f) (identical conditions as in Figure 2). (a, d) Luminescence spectroscopy (Eu^III^), (b, e) CD spectroscopy (La^III^), and (c, f) UV absorbance spectroscopy of Y96 (La^III^). Data from wt LanM (black lines) are reproduced for the sake of comparison.

The CD spectroscopy results were more intriguing (Figures 7b,e, S17b, and S18b). For both ΔEF1 and ΔEF2 variants, the CD signal at 222 nm saturates at 2 binding equivalents and at a lesser helicity than for the holo-wt protein, at both pH 7 and pH 5. This result suggests that alteration of the structures of EF1 and EF2 is most disruptive of LanM’s secondary structure. By contrast, the holo-ΔEF3 variant reaches the same helicity as the wt protein, but with a stoichiometry of ∼2, like the other variants, and with a less sharp transition between binding and saturation. Notably, however, apo-ΔEF3 is much more helical than apo-wt at pH 5, suggesting that the combination of the lower pH and the absence of some of the repulsive negative charges in the EF3 loop allows for partial preordering of a helical region near EF3. It is tempting to suggest that this preordered region may be the same part of the core helix α2 following EF3 that was highlighted by the NMR studies as having shifted resonances (and therefore some structural difference) at pH 5 and in K93A-LanM. Together, this would suggest that, in the wt protein, EF3 is indeed the third binding site, occupancy of which folds core helix α2.

The Y96 UV–visible titration data refine this interpretation (Figures 7c,f, S17c,d, and S18c,d). At pH 5, both ΔEF2 and ΔEF3 show significant absorbance at 287 nm even in the apoproteins, indicating that the preordering observed in the apo forms of these variants extends to both the secondary and tertiary structure. The pH 7 data is most revealing, however, with all variants showing markedly lower binding stoichiometry (∼2 equiv) than wt LanM and a maximum absorbance value that falls short of that of the wt protein by one-third. Even ΔEF3, in which the holoprotein possesses the same helical content as wt, fails to induce the full magnitude of change at 287 nm as in wt, denoting a perturbation of Y96′s hydrogen bond. As with wt LanM at pH 5, full secondary structure content seems to have been decoupled from tertiary structure.

Thus, the intact Y96-L131 hydrogen bond (“tertiary structure”) reported by UV spectroscopy is the most reliable readout to distinguish fully folded from incompletely folded protein, even when other methods (Eu luminescence, helical content by CD, apparent *K*_d_ values) seem to indicate “ideal” wt-like behavior. Of the different conditions examined – pH 5 and 7, comparison of wt, K93A-, and D56A-LanMs, substitutions in EF1, EF2, and EF3, and comparison of Ln^III^ ion vs Ca^II^ ion binding – the only one that yields the full absorbance change is the wt protein at pH 7. The small magnitude of chemical shift perturbations evident by comparing wt LanM NMR data at pH 5 and pH 7 (Figure 5b) highlights the subtlety of the transition induced by the third binding event.

## Discussion

Our results allow us to propose a refined model for cooperative Ln^III^ ion binding to LanM and the resulting protein conformational change (Figure 8). As in our previous model that proposed EF2/EF3 as the tightest-binding pair, the first step of our new, revised mechanism is the cooperative binding of two Ln^III^ ions, resulting in the majority (∼2/3) of LanM’s α-helix formation (from CD spectroscopy) as well as the formation of a tertiary fold (reported by Y96 absorption). Counter to those previous models, however, we have demonstrated the importance of EF1 for this cooperative binding. While our data do not definitively indicate which EF hand is the other half of this pair, they do suggest EF2 as a more likely candidate than EF3, and EF3 as the site of the third binding event. Furthermore, EF2 is directly connected to EF1 by a single α-helix (core helix α1), providing the most straightforward mechanism of communication between sites upon the first binding event. The lower absorbance signal from Y96, directly C-terminal to EF3, when only two ions are bound (evident in K93A-LanM, and in wt LanM at pH 5, for example), also suggests poorer packing of EF3 with the rest of the structure, implying that it may not be occupied under these conditions.

**Figure 8:**
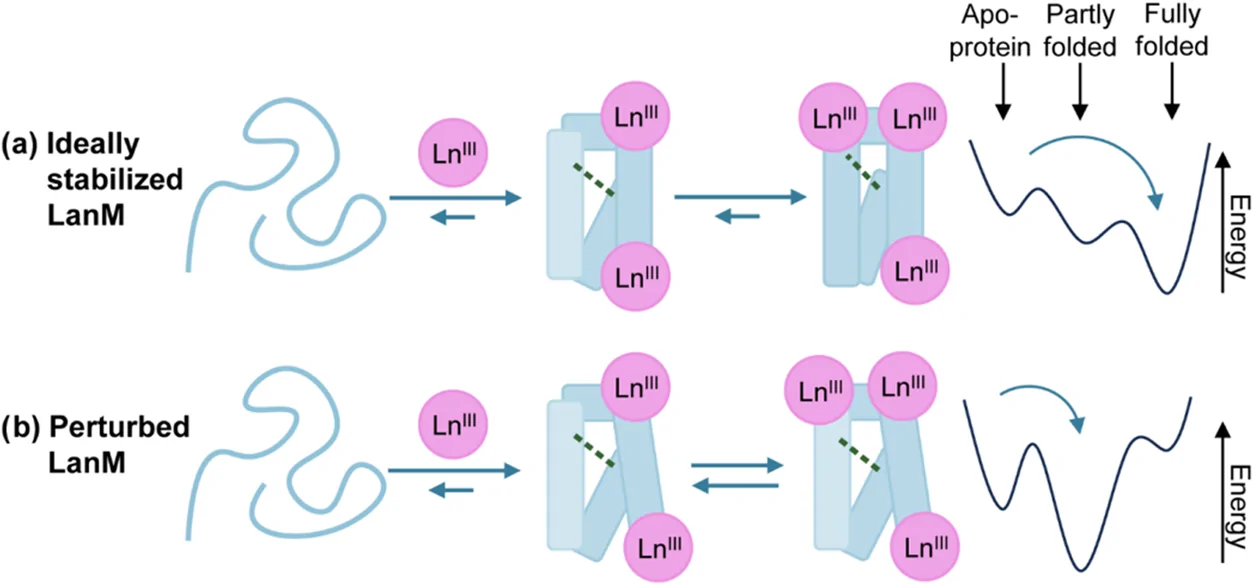
Cartoon representation of the proposed conformational exchange mechanism of LanM in response to Ln^III^ ions under (a) ideal conditions (preferred REEs, pH 7) and (b) conditions perturbed by pH, mutation, immobilization, or other means (e.g., non-REE binding). Energy wells illustrate the general (qualitative) thermodynamic driving forces that govern the population of each LanM structural conformation. Hydrogen bonding of Y96 is indicated by dashed green lines. The lighter color of helix α2 in the intermediate and perturbed states reflects our hypothesis that this helix only fully orders in the wt and at pH 7.

While the preponderance of evidence suggests that EF3 is the weaker site, we cannot distinguish whether EF1 or EF2 associates with an ion first, or how much secondary and tertiary structural formation accompanies the binding of the first ion. Nevertheless, the cooperativity observed for the initial pair implies some conformational ordering upon the first ion binding. The lower binding stoichiometry of the ΔEF1 variant might suggest that EF1 is of primary importance, and therefore may be the first (preferred) binding site, at least in vitro. It is easy to visualize how binding to EF1 could order both helix α1 and the C-terminal region via EF1′s interaction with EF4, thereby favoring binding to EF2. However, the available data do not exclude the possibility of EF2 being the site of the first binding event. In either case, the experimental results differ from those of a very recent purely computational study that suggested EF3 as the tightest site, which may reflect the limitations of the starting NMR structure (e.g., poorer definition of the carboxylate-rich coordination geometry than in the X-ray structures, and absence of K93 salt bridge), the difficulty of application of computational methods to REE-binding proteins, or unanticipated structural distinctions between yttrium and lanthanide binding.^47^

Although binding at the two high-affinity sites – and the resulting conformational change to a state which has significant secondary and tertiary structure – is strongly maintained at each condition tested and among all variants and conditions, the affinity of the protein for the third ion seems to be highly dependent on solution conditions (i.e., pH) and mutation. Based on results from NMR spectroscopy, this dependence seems to be linked to subtle conformational differences. Wild-type LanM at pH 7 has the highest affinity for the third Ln^III^ ion, while association is somewhat disfavored at pH 5, and greatly disfavored in the K93A variant, following the trend observed for perturbation to the core helices observed in the NMR spectra. At neutral pH, binding of the third ion is also tied to further ordering of the structure in wt LanM to the tightly packed state observed in the X-ray and NMR structures. The full extent of this conformational transition is not observed in any other condition; this is particularly apparent from Y96 absorbance data, in which no other sample reaches the peak intensity at 287 nm of wt LanM at pH 7.

What is the origin of the extreme sensitivity of the third event? Our data point to the importance of the salt bridge between D56 on core helix α1 and K93. We propose that the K93-D56 salt bridge forms upon occupancy of EF1 and EF2 and, K93 being the tenth residue in EF3, preorders the EF3 binding loop. Bringing the EF3 carboxylates into proximity through preordering might increase their p*K*_a_’s into a range where they can be protonated at mildly acidic pH values (e.g., pH 5), decreasing but not ablating the driving force for REE binding and lowering affinity at EF3. By contrast, the K93A substitution (or, in wt, protonation of D56 that might occur at even lower pH) would prohibit the preordering step and therefore interfere with binding. Such a mechanism would account for the spectroscopic data on the wt- and K93A-LanMs, as well as perhaps the differences observed between Ln^III^-bound and Ca^II^-bound LanM. Spectroscopic data indicate that the protein folds into a conformation similar to that of K93A-LanM, a result which may underlie the weak *K*_d_ of Ca^II^-bound LanM; decreased structural stability as compared to Ln^III^-bound LanM would be expected to make the ions more kinetically labile and has been suggested based on computational approaches.^48^

Notably, this hypothesis differs from our previous interpretation of experiments that seemed to suggest EF2/EF3 as the tight-binding EF-hand pair.^38^ That study utilized tryptophan residues installed into each EF hand to sensitize Tb^III^ luminescence, intended to probe the affinity of each EF-hand individually in order to assign binding order. This method suggested that EF2 and EF3 were the tightest binding, though at the time, we noted that some Trp substitutions within the EF-hands, especially in EF2 and EF3, disrupted conformational response or reduced cooperativity. We likely underappreciated the impact of the altered cooperativity on our interpretation. We also lacked a definitive probe of EF2; K94W, the least perturbative probe, was assumed to report on EF3, but, given the proximity of EF2 and EF3, which site was being reported on was not known without question. The present work reiterates that subtle substitutions can have large impacts on cooperativity and structure in LanM; indeed, the ease of such perturbation could provide a mechanism for the evolution of an altered metal-binding order in different LanMs. For example, *Hans*-LanM lacks the K93-D56 salt bridge but possesses other interhelical and interchain interactions, and EF3 is integral for dimerization;^12^ therefore, its metal-binding order may be distinct from that of *Mex*-LanM.

Our observations regarding the facile inactivation of the third binding event and its localization to EF3 have implications for the application of LanM in separations. LanM immobilized to agarose microbeads^14^ (or other substrates^15,19,20,49,50^ ) via a short C-terminal linker has been applied for REE recovery and separations. Unlike the protein in solution, which binds the third equivalent weakly at pH 5, the immobilized protein binds only two REEs.^14^ While decreased stoichiometry could result from steric limitations related to the immobilization, our results suggest that it is more likely that C-terminal perturbation via immobilization is sufficient to disfavor binding of a third Ln^III^ ion through conformational effects, i.e., by disallowing the protein from reaching the optimal tightly packed condition under which the third Ln^III^ ion equivalent binds cooperatively. LanM’s C-terminus is located very close to the junction of EF2 and EF3, to Y96 and to the K93-D56 salt bridge, and the C-terminal carboxylate is engaged in salt bridges with R74 and K57. Thus, perturbation at the C-terminus may have similar impacts to the perturbations studied herein, either independently or by altering core interactions such as the K93-D56 salt bridge.

Such conformational effects may also explain why C-terminally immobilized LanM exhibits somewhat greater REE/REE selectivity than the protein in solution,^10,22^ suggesting links between LanM’s tertiary structure and REE/REE selectivity, not just tertiary structure and high affinity.^51^ REE selectivity profiles for most well-characterized protein/peptide-based ligands – lanthanide binding tags (LBTs),^52^ conventional Ca^II^-binding EF-hand proteins,^53^ isolated LanM EF-hand peptides,^24^ native REE-binding proteins without EF-hands,^42^ and biomimetic REE-transporting channels^30^ – mirror water exchange rates, with maximum affinity/transport rates around Eu, decreasing sharply toward lighter REEs and more gradually toward heavy REEs.^54^ LanMs diverge from this profile in terms of both affinity maxima and steepness of their selectivities on either side, with different LanMs doing so to differing extents.^11,12^ With the exception of *Hans*-LanM and *Al*-LanM, where protein dimerization is involved in shifting REE/REE selectivity, the mechanisms by which tertiary structure influences selectivity are poorly understood but will be essential for design – rather than mere reversion to a nonspecific selectivity profile – of proteins with non-native selectivity for separation applications.

Our results also provide an explanation for the (at the time) unexpected behavior of a size-minimized LanM (EF2/3) comprising EF2, EF3, and the residues between them.^23^ Based on our earlier model, we had anticipated that linking these two EF-hands would yield more wt-like coordination (2 solvent molecules) and higher affinity than single EF-hands, which have 5–6 water molecules and only micromolar affinity.^24,55^ Surprisingly, our results suggested only one of the EF-hands was competent to bind, and only with micromolar affinity, although the coordination environment was partially rescued. The present results rationalize the EF2/3 results by demonstrating the importance of EF1 in maintaining high-affinity cooperative binding in LanM as well as the importance of the K93-D56 salt bridge to activate EF3 for binding. Thus, our work indicates a new strategy for the design of size-minimized LanMs.

Finally, this model also supports our current understanding of LanM’s role in periplasmic Ln^III^ ion transport. *Mex*-LanM is a part of a ten-gene cluster encoding proteins with putative roles in Ln^III^ import and trafficking. LanM interacts with another small Ln^III^-binding protein, landiscernin (LanD), and evidence suggests that LanM receives Ln^III^ ions from LanD, as part of a pathway to control selectivity among REEs in uptake and utilization.^42^ Because LanD binds only one Ln^III^ ion, it follows that LanM receives one ion at a time. Thus, a mechanism in which a single, high-affinity binding event is sufficient to fold and template the protein for binding of additional Ln^III^ ion equivalents would be physiologically advantageous. Studies of the LanD-LanM complex are underway, and we anticipate that they will allow us to better understand the role of ordered Ln^III^ ion binding in LanM.

## Conclusion

We present a revised model for the mechanism of Ln^III^ ion binding and associated conformational change in *Mex*-LanM. Contrary to previous models, our data strongly suggest that the initial tight-binding cooperative EF hand pair is EF1/EF2 and is associated with LanM’s major conformational change. We also define the interhelix K93-D56 salt bridge as a crucial determinant for binding of the third metal ion, likely at EF3, underscoring how protein structure and interactions outside the EF-hands impact Ln^III^-binding cooperativity and stoichiometry in LanM. Armed with the definition of conditions and variants that might stabilize partially folded intermediates, ongoing work will elucidate this model in higher resolution, in terms of kinetics, enthalpic and entropic contributions, and structure. These results have great importance for the ongoing development of strategies for the recovery and separation of Ln^III^ ions using, or inspired by, LanM proteins. Such applications rely on an accurate understanding of the positions at which Ln^III^ ions bind and the minimal protein interactions required to maintain the native properties of LanM, which to this point had been obscure. Our results, therefore, provide a valuable foundation on which the design of LanM variants and other REE-binding proteins with novel properties may be based.

## Supplementary Material



## Data Availability

All data supporting this article are included in the manuscript and/or the Supporting Information.

## Notes

Additional experimental details, materials, and methods; additional figures including absorbance, fluorescence, circular dichroism, and NMR spectra. (PDF)
