# Supplementary material for: EF-Hand Binding Order and Conformational Control of Lanthanide Ion Binding in M. extorquens Lanmodulin
Source: Biochemistry. 2026 Oct 13;65(21):3501–13. doi: 10.1021/acs.biochem.6c00566 (PMC13738343; doi:10.1021/acs.biochem.6c00566)
Supplement: Supplementary file 1 [file bi6c00566_si_001.pdf]

Supporting Information for

EF-hand binding order and conformational control of lanthanide ion binding in *M. extorquens* lanmodulin

Claire C. Mammoser, Natalie A. Savastio, Joseph A. Cotruvo, Jr.*

Department of Chemistry, The Pennsylvania State University, University Park, Pennsylvania, United States

*Corresponding author: juc96@psu.edu

This file contains the Supplementary Methods, Supplementary Table S1, Supplementary Figures S1-S18, and Supplementary References.

Supplementary Methods **Page S3**

General considerations
Expression and purification of wt and variant LanMs
Stoichiometric titrations monitored using Eu^{III} luminescence
Stoichiometric titrations monitored using CD spectroscopy
Stoichiometric titrations monitored using UV absorbance spectroscopy
Determination of K_D using UV absorbance spectroscopy of chelator-buffered solutions
Expression of ^{15}N-labeled wt and K93A LanM
NMR spectroscopy of $[^1H,^{15}N]$- LanM
Determination of LanM's tight-binding stoichiometry by competition with xylenol orange
Determination of LanM's tight-binding stoichiometry by spin desalting

Supplementary Table **Page S11**

Table S1. Tight-binding stoichiometries determined by spin column assay

Supplementary Figures **Page S12**

Fig. S1. Representative spectra from stoichiometric titrations of wt and K93A LanM
Fig. S2. Absorbance response of Y96 in K93A LanM at pH 5.0
Fig. S3. Spectroscopic response to stoichiometric binding of Ln^{III} ions in D56A LanM
Fig. S4. Representative spectra from stoichiometric titrations of D56A LanM
Fig. S5. La^{III}-LanM affinity titrations (wt, K93A, and D56A) at pH 7.0 and pH 5.0
Fig. S6. Spectra from titrations of wt LanM with Ca^{II} at pH 7.0
Fig. S7. Absorbance response of Y96 in wt LanM at pH 7.0 to binding of Ln^{III} ions
Fig. S8. NMR spectra of wt LanM at pH 7.0 loaded with 3 equiv. Y^{III}
Fig. S9. NMR spectra of wt LanM at pH 7.0 loaded with La^{III} and with Y^{III}
Fig. S10. NMR spectra of La^{III}-bound wt (3 equiv.) and K93A (2 equiv.) LanM at pH 7.0
Fig. S11. NMR spectra of La^{III}-bound wt LanM at pH 7.0 (3 equiv.) and at pH 5.0 (2 equiv.)
Fig. S12. NMR spectra of Y^{III}-bound wt (3 equiv.) and K93A (2 equiv.) LanM at pH 7.0
Fig. S13. NMR spectra of Y^{III}-bound wt LanM at pH 7.0 (3 equiv.) and at pH 5.0 (2 equiv.)
Fig. S14. Chemical shift perturbation values of LanM plotted by residue number
Fig. S15. CD spectra showing impact of storage on conformational response of ΔEF1 LanM
Fig. S16. La^{III}-LanM affinity titrations ($D_{3,5,9}N$ variants) at pH 7.0 and pH 5.0
Fig. S17. Representative spectra from stoichiometric titrations of $D_{3,5,9}N$ LanM at pH 7.0
Fig. S18. Representative spectra from stoichiometric titrations of $D_{3,5,9}N$ LanM at pH 5.0

Supplementary References **Page S28**

SUPPLEMENTARY METHODS

General considerations. General chemical reagents were acquired from Millipore Sigma unless otherwise noted. Lanthanide (Ln^{III}) chloride salts were obtained at 99.9% purity of the metal ion content and stock solutions were prepared in 1 M HCl to approximately 0.5 M Ln^{III} ion concentration. Exact concentrations of the Ln^{III} stock solutions were determined by inductively coupled plasma mass spectrometry (ICP-MS, Thermo Scientific iCAP TQe) in the Pennsylvania State University Laboratory for Isotopes and Metals in the Environment. BL21(DE3) *E. coli*, used for all protein expression, was obtained from New England Biolabs (Ipswich, MA). All plasmids for expression of LanM variants were purchased from Twist Bioscience in pET-29b(+) vectors (San Francisco, CA). Chromatography for LanM purification was performed using Q-sepharose Fast Flow resin (Millipore Sigma) and a HiLoad 16/600 Superdex 75 pg column (Cytiva, Wilmington, DE).

Absorbance spectra were acquired using a Cary 60 UV-visible spectrometer (Agilent, Santa Clara CA). For stoichiometric titrations and determination of protein concentrations, a quartz cuvette with 10 mm (path length) × 2 mm dimensions (18B-Q-10, Starna Cells, Inc., Atascadero, CA) was used; for determinations of Ln^{III}-protein affinities, a 10 mm path length quartz cuvette designed for use with <100 μL samples (16.50-Q-10/Z20, Starna Cells, Inc.) was used. Protein concentrations were calculated based on $\varepsilon_{280} = 1490\ M^{-1}\ cm^{-1}$. Luminescence spectra were acquired on a Horiba Fluorolog-QM spectrometer in configuration 75-21-C using a Xenon flash lamp in a quartz cuvette with 10 mm (path length) × 2 mm dimensions (Starna Cells, Inc.). CD spectra were acquired using a Jasco J1500 CD spectrometer equipped with a Xenon arc lamp and double monochromator in the Penn State Biomolecular Interaction Facility, using a 1 mm path length quartz cuvette (21-Q-1, Starna Cells, Inc.). NMR spectra were collected on a Bruker NEO-2 600 MHz NMR spectrometer at the High-Resolution NMR Facility for Research and Education at Penn State.

For all titrations, LanM (wt or variant) aliquots were thawed and stored at 4 °C for less than one week while in use. While no impact from storage over this period was observed in most samples, certain conditions and experimental methods showed particular sensitivity to the storage time of the protein. Specifically, circular dichroism spectra of wt LanM at pH 5 showed a decreased magnitude of the change in molar ellipticity as the incubation time increased, with some titrations

collected using longer-stored protein resembling those of the K93A-LanM variant (sensitivity to storage time was not observed using other spectroscopic methods under the same conditions). As mentioned in the main text, the ΔEF1 $D_{3,5,9}N$ variant was particularly susceptible to deviation in its spectral responses to metal ion binding over time. For this reason, special care was taken to collect data within 2-3 days of thawing, at which time these deviations were not evident.

Expression and purification of wt and variant LanMs. Expression and purification of LanM used similar procedures to our previous reports;[1, 2] the same procedure was followed for wt LanM (*Mex*-LanM) and all variants. Plasmids encoding the genes for LanM variants were purchased from Twist Bioscience (San Francisco, CA). Genes were expressed from a pET-29b(+) vector under an IPTG-inducible promoter, and the N-terminal signal peptide sequence was omitted, resulting in cytoplasmic protein expression. Expression plasmids were transformed into chemically competent BL21(DE3) *E. coli* and plated on LB-agar plates containing 50 μg/mL kanamycin (GoldBio, St. Louis, MO). A single colony was used to inoculate a 100 mL culture of LB media containing 50 μg/mL kanamycin and the culture was allowed to grow overnight at 37 °C with 200 rpm orbital shaking. Following ~14-16 h incubation, 40 mL of this culture was used to inoculate 2 L of LB media containing 50 μg/mL kanamycin in a 6 L flask. This culture was incubated at 37 °C with 170 rpm orbital shaking until the OD_{600} of the cells was 0.6-0.8, at which point protein expression was induced by addition of 200 μM isopropyl β-D-thiogalactopyranoside (IPTG, Oakwood Chemical, Estill, SC). Expression was allowed to proceed for 3-4 h under the same incubation conditions, following which the cells were harvested by centrifugation at 7,000 ×*g* for 10 min. Harvested cells were stored at -80 °C.

For cell lysis and protein purification, cells were thawed on ice and resuspended by serological pipette in 5 mL/g of cell pellet 50 mM Tris pH 8.0, 10 mM NaCl, 5% glycerol. One protease tablet (Roche cOmplete) per 35 mL resuspension and 2 units/mL resuspension DNAse (New England Biolabs, Ipswich, MA) were added. Cells were lysed by sonication (QSonica, Newtown, CT) at 50% amplitude using pulses of 5 s on, 8 s off for a total of 12 min. Lysate was clarified by centrifugation at 40,000 ×*g* for 35 min. Clarified lysate was loaded to a Q-sepharose column equilibrated in the lysis buffer. After washing with the lysis buffer, the protein was eluted by a linear gradient to 50 mM Tris pH 8.0, 600 mM NaCl, 5% glycerol. Fractions were evaluated by SDS-PAGE electrophoresis and fractions with significant LanM content were pooled and

concentrated to <5 mL. Protein was loaded to a HiLoad 16/600 Superdex 75 pg column (Cytiva, Wilmington, DE) equilibrated in 30 mM MOPS pH 7.0, 100 mM KCl, 5 mM $CaCl_2$, 5% glycerol. The column was run using the same buffer at a flow rate of 1 mL/min using an Akta Pure fast protein liquid chromatography system at 4 °C (Cytiva, Wilmington, DE). Fractions containing pure LanM were pooled, concentrated to ~10 mL, and dialyzed against 30 mM MOPS pH 7.0, 100 mM KCl, 5% glycerol with 1 g/100 mL Chelex-100. Protein was flash frozen in liquid N_2 and stored in the same buffer at -80 °C. Typical yields were >30 mg LanM per L culture for wt and variant LanMs.

Stoichiometric titrations monitored using Eu^{III} luminescence. In a 1 cm path length quartz cuvette, 600 μL of 20 μM LanM (wt or variant) was prepared in 30 mM HEPES pH 7.0, 100 mM KCl treated with Chelex-100. The concentration of a stock solution of Eu^{III} in 1 M HCl was determined using ICP-MS analysis; from this stock, a 3 mM working solution was prepared in the same buffer. The titration was performed by 1 μL (5 μM) additions to the cuvette from this working solution; the solution in the cuvette was mixed by pipetting following each addition.

Spectra were acquired on a Horiba Fluorolog-QM spectrometer in configuration 75-21-C using a Xenon flash lamp with 394 nm excitation (7 nm excitation slit width, 15 nm emission slit width), 150 μs collection delay time, 1100 μs collection end time. Data was collected from 560-650 nm at a 0.5 nm step size, averaging 100 shots per step. Eu^{III} luminescence spectroscopy gives two major emission peaks at 595 and 619 nm. These peaks were fitted to a superposition of two Gaussians, and the maximum intensity of the fit to the peak at 619 nm, which shows a stronger response to coordination, was plotted as a function of Eu^{III} added. Data shown represent the mean and standard deviation from three independently acquired titrations.

Stoichiometric titrations monitored using CD spectroscopy. In a 0.1 cm path length quartz cuvette, 350 μL of 20 μM LanM (wt or variant) was prepared in 30 mM MOPS pH 7.0, 100 mM KCl treated with Chelex-100. A 1.75 mM working solution of La^{III} was prepared from an ICP-MS-standardized stock as described for stoichiometric titration with Eu^{III}. The titration was performed by 1 μL (5 μM) additions of La^{III} into the cuvette, mixing following each addition.

Spectra were collected at 20 °C using a Jasco J1500 CD spectrometer equipped with a Xenon arc lamp and double monochromator in the Penn State Biomolecular Interaction Facility.

Data were collected from 195-255 nm with a step size of 0.5 nm. Signal at 222 nm was converted to molar ellipticity (deg cm^2 $dmol^{-1}$) and plotted as a function of La^{III} added; each point represents a mean from three independently acquired titrations.

Stoichiometric titrations monitored using UV absorbance spectroscopy. In a 1 cm path length quartz cuvette, 600 µL of 20 µM LanM (wt or variant) was prepared in 30 mM HEPES pH 7.0, 100 mM KCl treated with Chelex-100. The concentration of a stock solution of La^{III} in 1 M HCl was determined using ICP-MS analysis; from this stock, a 3 mM working solution was prepared in the same buffer. The titration was performed by 1 µL (5 µM) additions to the cuvette from this working solution; the solution in the cuvette was mixed following each addition.

Spectra were collected from 240-340 nm with a step size of 0.5 nm and averaging time of 0.05 s using a Cary 60 UV-visible spectrometer (Agilent, Santa Clara, CA). Due to the low extinction coefficient of LanM, the absorbance intensity at 287 nm (corresponding to hydrogen binding of Tyr96) was determined as the intensity of the signal relative to a baseline drawn between 283 and 291 nm of the same spectrum (dropline correction). Dropline absorbance at 287 nm was plotted as a function of La^{III} added. Data shown represent the mean of three replicate titrations; error bars represent the standard deviation of the replicates for each point.

Determination of K_D using UV absorbance spectroscopy of chelator-buffered solutions. Due to the high affinity of LanM for Ln^{III} ions, apparent dissociation constants (K_Ds) for the tight-binding Ln^{III} ion equivalents to LanM were determined using solutions of La^{III} buffered by a chelator as described previously.[1, 3] To summarize, La^{III} was standardized against chelators EGTA (for use at pH 7) and DTPA (for use at pH 5) using xylenol orange (XO) indicator by the following method. Solutions of approximately 1 M La^{III} in 1 M HCl and 0.5 M chelator were used to prepare solutions of nominal 10 mM concentration in 30 mM MES pH 6.0, 100 mM KCl. In a quartz cuvette, 25 µL of the 10 mM chelator solution and 0.5 µL of 5 mM XO were added to 470 µL of the same buffer. UV-visible spectra were acquired from 350-800 nm, tracking the absorbance of XO at 575 nm, which increases upon Ln^{III} coordination. La^{III} was added to the cuvette from the 10 mM solution, mixing after each addition. Since the affinity of XO for La^{III} is substantially lower than that of EGTA for La^{III}, no change in the UV-vis spectrum is observed upon addition of La^{III} from the 10 mM stock until all chelator is associated with La^{III}; the equivalence point for the La^{III}-

chelator pair is therefore considered to be at the first addition of La^{III} resulting in an increase in absorbance at 575 nm.

Buffers containing chelator alone (nominally 10 mM) and 1:1 chelator:La^{III} based on the results of the standardization were prepared from 1 M La^{III} and 0.5 M chelator stock solutions in 30 mM MOPS, 100 mM KCL (pH 7.0) or 30 mM acetate, 100 mM KCl (pH 5.0) and adjusted to the appropriate pH. LanM (wt or variant) was diluted from a high-concentration stock to a final concentration of 15 μM in each of the buffers. An effective K_D ($K_{D,M}$) for the chelator-metal pair under the experimental conditions was calculated using Eq. 1, where K_M is the standard stability constant of the chelator-metal pair and pK_a values are proton association constants for the chelator taken from Martell and Smith.[4] Different ratios of LanM in the chelator-only and 1:1 chelator:La^{III} buffers were mixed to yield solutions of protein with a range of free La^{III} (M_{free}) determined by Eq. 2. Each 120 μL solution was incubated overnight to allow for equilibration due to slow kinetics of La^{III} exchange with the chelators.

$$K_{D,M} = \frac{1+10^{pKa_1-pH}+10^{pKa_1+pKa_2-2pH}+10^{pKa_1+pKa_2+pKa_3-3pH}}{10^{K_M}} \quad \text{Eq. 1}$$

$$[M_{free}] = K_{D,M}\frac{[ML]}{[L]} \quad \text{Eq. 2}$$

UV absorbance spectra were acquired with the same parameters as for stoichiometric titrations (240-340 nm, 0.5 nm step size, 0.05 s averaging time). Absorbance intensity at 287 nm was determined through the dropline correction method (baseline drawn between 283 and 291 nm) as described for stoichiometric titrations. Absorbance values were plotted against calculated values for free La^{III} and fitted to the Hill equation (MATLAB 2019b). Reported values for K_D and Hill coefficient (n) are the mean values from three independent replicates. Plotted curves show the mean and standard deviation of three replicate data points at each free La^{III} concentration; the fit shown is calculated based on the mean value for each point.

Expression of ^{15}N-labeled wt and K93A LanM. ^{15}N-labeled LanM (wt and K93A) was expressed in defined media following the same general procedure as described for expression of unlabeled proteins. Defined media was prepared by dissolving 3 g/L monobasic potassium phosphate, 6 g/L dibasic potassium phosphate, and 0.5 g/L NaCl in 18 MΩ water (typical expression volume is 25 mL for starter culture, 500 mL for expression culture). Following sterilization of the culture media by autoclave, glucose (final concentration 4 g/L) and $MgSO_4$ (final concentration 400 μM) were

added to the culture from 50× filter-sterilized stock solutions. At the time of inoculation, the media was additionally supplemented with 1.0 μg/mL thiamine, 0.5 μg/mL riboflavin, 1.0 μg/mL biotin, 5 ng/mL lipoic acid, trace metals (50 μM $FeCl_3$, 50 μM $CaCl_2$, 10 μM $MnCl_2$, 10 μM $ZnCl_2$), 50 μg/mL kanamycin, and 1 g/L $^{15}NH_3Cl$ (99% ^{15}N, Cambridge Isotope Laboratories, Tewksbury, MA). Conditions and methods for the expression and purification of ^{15}N LanM proceeded as described for the unlabeled proteins.

NMR spectroscopy of [^1H,^{15}N]-LanM. Buffers for NMR analysis of La^{III}- or Y^{III}-bound LanM were prepared using either 30 mM MOPS pH 7.0, 100 mM KCl; or 30 mM acetate pH 5.0, 100 mM KCl as a base. To 10 mM of the base buffer La^{III} or Y^{III} from ICP-standardized stock solutions was added based on the desired number of ion equivalents relative to LanM (2 equiv. at pH 5.0 and for K93A LanM at pH 7.0, 3 equiv. for wt LanM at pH 7.0) and final protein concentration. For example, for a sample of wt LanM at pH 5.0 at a final concentration of 300 μM (333 μM before cosolvent addition), two equivalents (666 μM) were added by dilution from the La^{III} or Y^{III} stock. These buffers were prepared immediately prior to use to reduce the impact of hydrolysis of the ions. The pH of the buffer was adjusted following addition of the rare earth stock solution, as addition significantly lowers the pH. LanM was then exchanged into the REE ion-containing buffer using 500 μL centrifugal spin filters (Amicon). Protein (450 μL) was first loaded to the filter and concentrated at 15,000 ×*g* to approximately 150 μL. 300 μL of the REE ion-containing buffer was added, and the centrifugal concentration was repeated. This process was repeated through four exchanges, resulting in a concentrated protein sample in >98% REE-containing buffer. The protein was then diluted in the same buffer to the concentration used to determine the amount of REE ion added (in the above example, 333 μM), to yield 450 μL LanM with the desired ion stoichiometry. To each sample was added 10% (v/v) D_2O (TCI, Portland, OR) as a lock co-solvent. Final samples were 500 μL with LanM concentrations from 300-500 μM. The absence of apoprotein peaks in the NMR spectrum supports the added 2 equiv. of metal ions being fully bound to the protein at pH 5.

^1H, ^{15}N heteronuclear single quantum coherence (HSQC) spectra were collected at 25 °C on a Bruker NEO-2 600 MHz NMR spectrometer at the High-Resolution NMR Facility for Research and Education at Penn State. Data were collected using TopSpin 4.4 (Bruker, Billerica, MA) and analyzed using NMRFAM Sparky.[5]

Backbone resonances were assigned to residues of wt LanM by comparison with previously reported spectra of the Y^{III}-bound protein at pH 7.0.[6] Resonances in spectra acquired under other conditions were assigned based on comparison with assignment of this spectrum. Backbone chemical shift perturbations (Δδ) were determined using the formula, $\Delta\delta = [(\Delta\delta H)^2+0.2(\Delta\delta N)^2]^{1/2}$. Chemical shift perturbation values assigned to the backbone of each residue were sorted based on their magnitude and assigned to a logarithmic color scale which was used to indicate the position and magnitude of the perturbation on a structural model of the protein.

Determination of LanM's tight-binding stoichiometry by competition with xylenol orange. A solution of 500 μM xylenol orange (XO) was prepared in 30 mM MES pH 6.0, 100 mM KCl. A solution of La^{III} (1.2 mM) was prepared from an ICP-MS-standardized stock solution in the same buffer. In a 1 cm path length quartz cuvette, 600 μL of 5 μM XO and 10 μM wt or variant LanM was prepared in the same buffer. Using a Cary 60 UV-visible spectrometer (Agilent, Santa Clara, CA), a spectrum of the initial solution was acquired (300-800 nm, 1 nm step size, 0.0125 s averaging time). 1 μL (2 μM) of the 1.2 mM La^{III} stock was added and mixed well prior to each subsequent spectral acquisition; the titration continued until the signal at 575 nm, indicative of La^{III}-bound XO, had saturated. Absorbance at 575 nm was plotted against the number of equivalents of La^{III} added. Data shown are the mean of three independent titrations; error bars represent the standard deviation of the replicates for each point.

Determination of LanM's tight-binding stoichiometry by spin desalting. Zeba spin desalting columns (0.5 mL, ThermoFisher) were equilibrated into 30 mM HEPES, pH 7.0, 100 mM KCl by first centrifuging the column at 5000 ×*g* in a microcentrifuge for 1 min to remove the storage solution, then adding 300 μL buffer to the resin, and centrifuging it out under the same conditions. The same buffer addition and centrifugation procedure was repeated a total of six times.

LanM (130 μL, 20 μM) with 80 μM (4 equivalents) La^{III} was prepared in the above buffer, and incubated for a minimum of 20 min. Following equilibration of the spin column, the protein solution was loaded onto a column equilibrated in the same buffer. Protein was eluted from the column by centrifugation under the same conditions as for equilibration; LanM and any bound ions which are not kinetically labile eluted from the column, while ions not tightly bound to the protein remained in the column.

A portion of the eluate from the spin column was diluted 100-fold into 2% trace metals-free nitric acid and analyzed by ICP-MS (Thermo Scientific iCAP TQe, Penn State Laboratory for Isotopes and Metals in the Environment). Protein concentrations were determined by Pierce BCA protein assay (ThermoFisher) using a linear fit to a set of LanM standards from 0-250 µg/mL protein. From the La^{III} concentrations and protein concentrations determined through these methods, La^{III} stoichiometry was determined for each LanM sample.

SUPPLEMENTARY TABLE

Table S1. Tight-binding stoichiometry data for La^{III} binding to LanM variants (20 μM) determined by ICP-MS of spin desalting column eluates. ND = not determined. pH 5 data are also presented in **Figure 6b**.

	**pH 5 (ions per LanM)**	**pH 7 (ions per LanM)**
wt	2.5 ± 0.3	2.8 ± 0.2[a]
K93A	ND	1.6 ± 0.2
D56A	ND	1.7 ± 0.3
ΔEF1	1.4 ± 0.2	1.2 ± 0.2[a]
ΔEF2	2.1 ± 0.2	2.2 ± 0.3[a]
ΔEF3	2.3 ± 0.2	2.4 ± 0.2[a]

[a] reported data represents the average and standard deviation of two replicate samples; all other data represents the average and standard deviation of three replicate samples. Each data point represents an individually prepared spin desalting column eluate.

SUPPLEMENTARY FIGURES

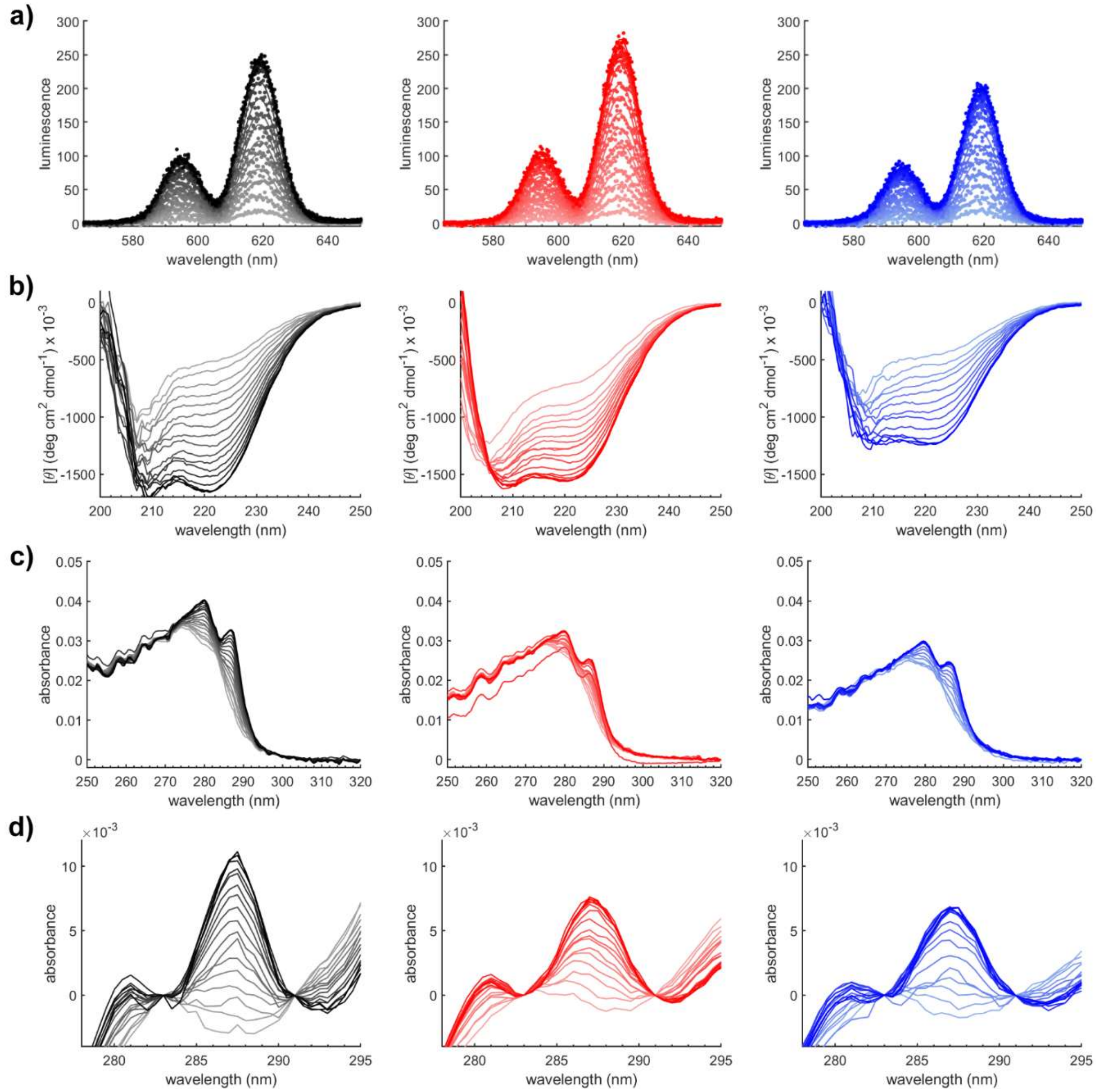

Figure S1. Spectra from stoichiometric titrations of LanM (20 μM; left, wt at pH 7; center, wt at pH 5; right, K93A at pH 7). Darker lines indicate more Ln^{III} added. Spectra were acquired by: **a)** titration with Eu^{III} measured by luminescence spectroscopy with excitation at 394 nm; **b)** titration with La^{III} measured by circular dichroism spectroscopy; **c)** titration with La^{III} measured by UV-visible spectroscopy. **d)** shows the spectra in **c)** corrected by subtraction of a linear baseline between the absorbances at 283 and 291 nm. Spectra shown are from representative titrations; each stoichiometric determination was an average of three replicate experiments.

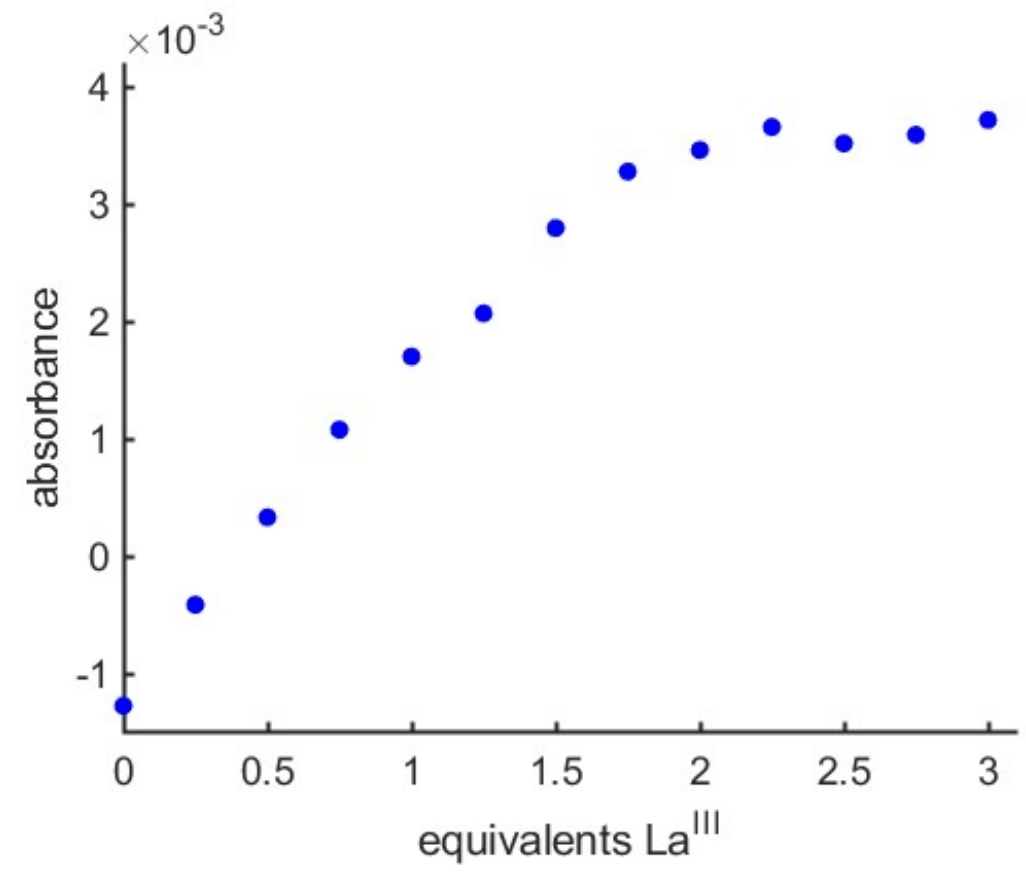

Figure S2. Stoichiometric titration of K93A LanM (20 µM) with La^{III} at pH 5.0, followed by Y96 absorbance (287 nm). Data shown are results of a single titration.

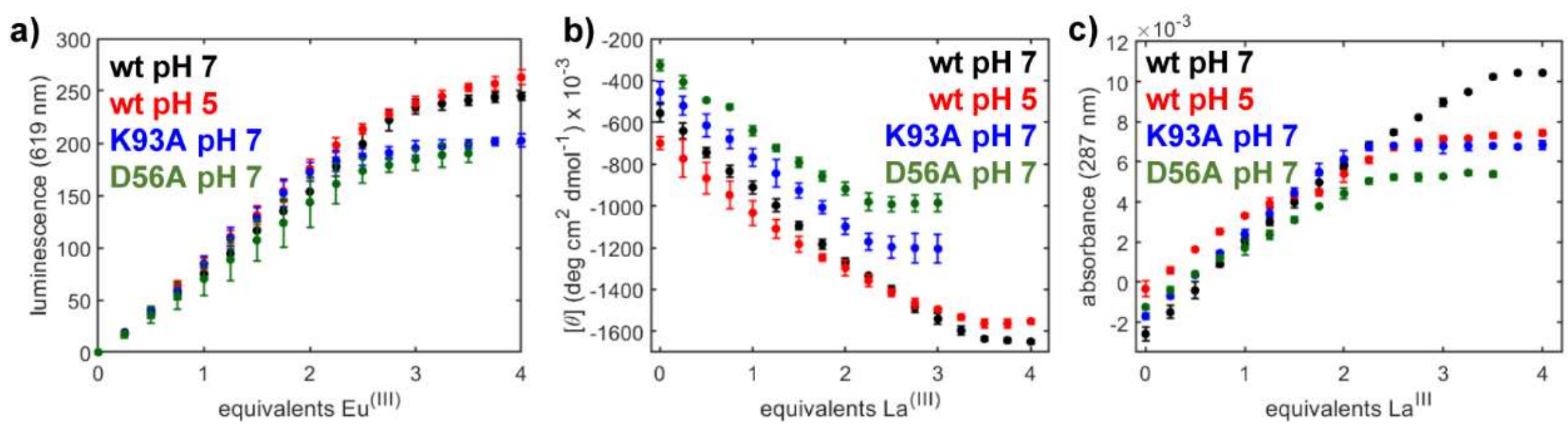

Figure S3. Spectroscopic response to binding of Ln^{III} ions to wt, K93A, and D56A LanM. **a)** Luminescence spectroscopy (Eu^{III}), **b)** CD spectroscopy (La^{III}), and **c)** UV absorbance spectroscopy of Y96 (La^{III}). In **(c)**, values reported result from subtraction of a linear baseline calculated between 283 and 291 nm, isolating the absorbance related to hydrogen bonding of Y96 from the underlying tyrosine absorbance. Protein concentrations are 20 µM for all experiments. Error bars indicate the standard deviation from triplicate measurements of separate samples.

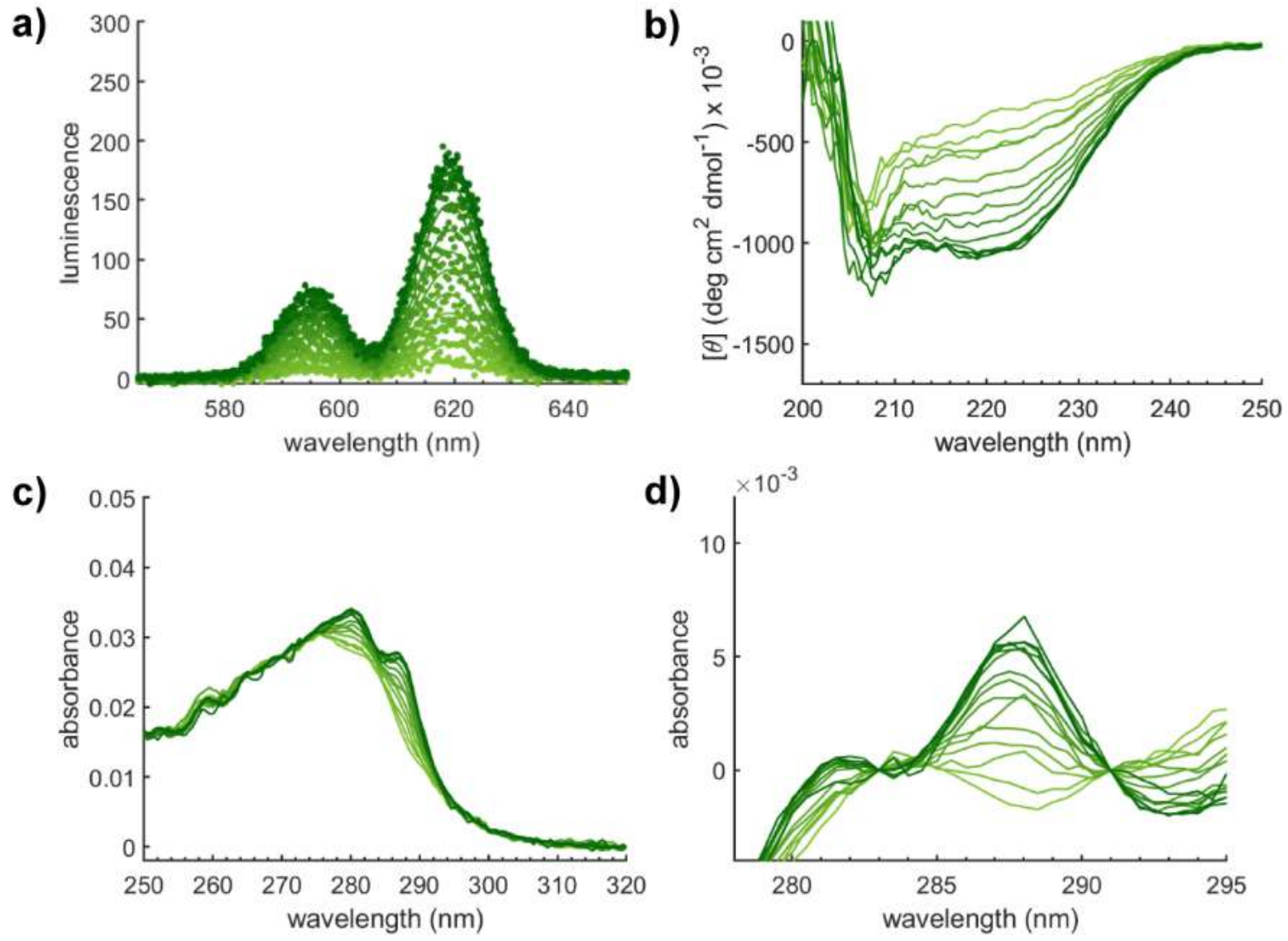

Figure S4. Spectra from stoichiometric titrations of D56A LanM at pH 7 (20 μM). Darker lines indicate more Ln^{III} added. Spectra were acquired by: **a)** titration with Eu^{III} measured by luminescence spectroscopy with excitation at 394 nm; **b)** titration with La^{III} measured by circular dichroism spectroscopy; and **c)** titration with La^{III} measured by UV-visible spectroscopy. **(d)** The spectra in **(c)** were corrected by subtraction of a linear baseline between the absorbances at 283 and 291 nm. Spectra shown are from representative titrations; each stoichiometric determination was an average of three replicate experiments.

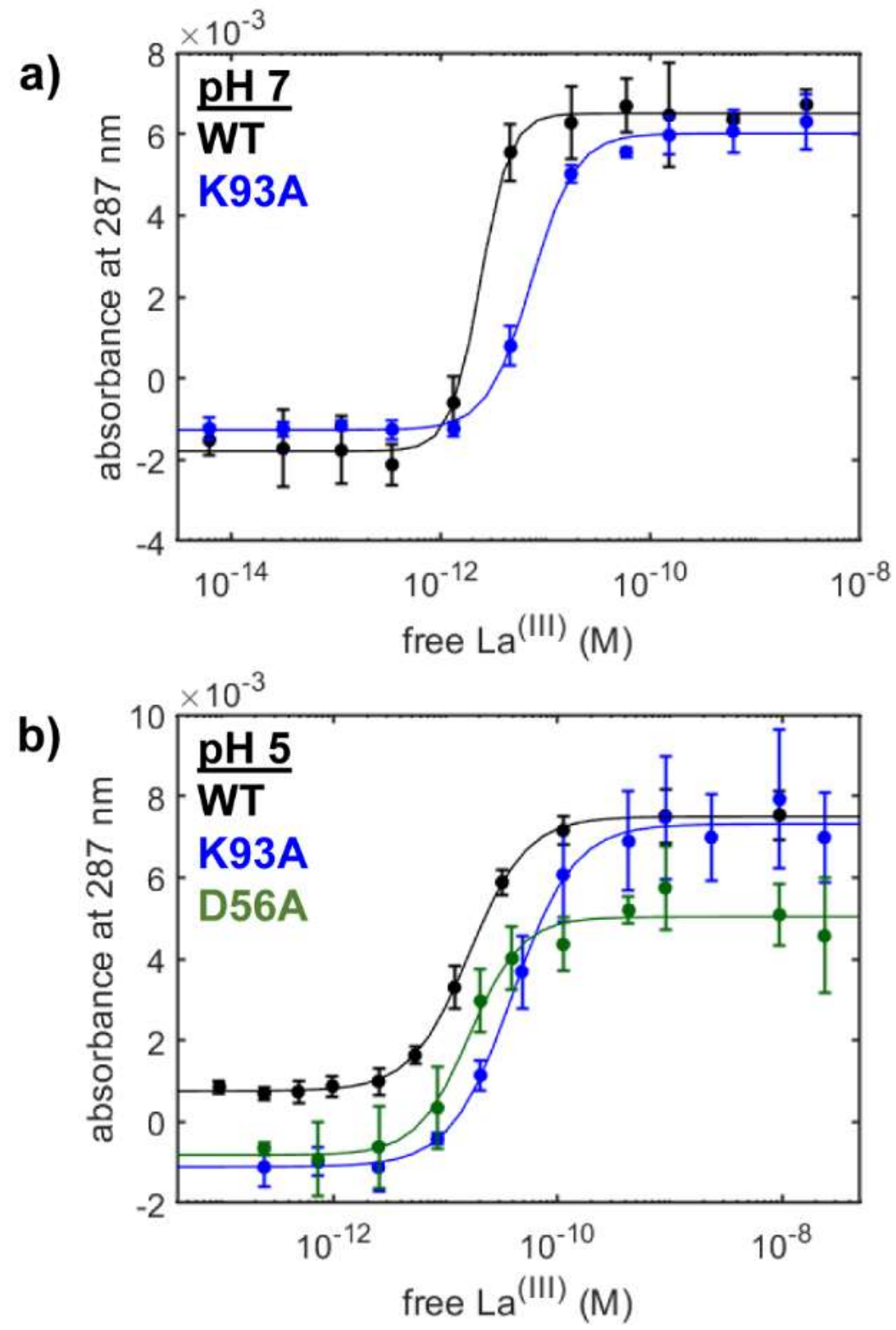

Figure S5. UV absorbance response of Y96 in LanMs (15 μM) to binding La^{III}, monitored at 287 nm, to determine affinity (K_D) at **a)** pH 7 (wt, K93A) and **b)** pH 5 (wt, K93A, D56A). Solid lines represent fits of the data to the Hill equation. Note that the absolute responses are smaller than those shown in the main text (**Figs. 2 and 3**) because of the lower concentration (15 vs. 20 μM). Error bars indicate the standard deviation from triplicate measurements of separate samples.

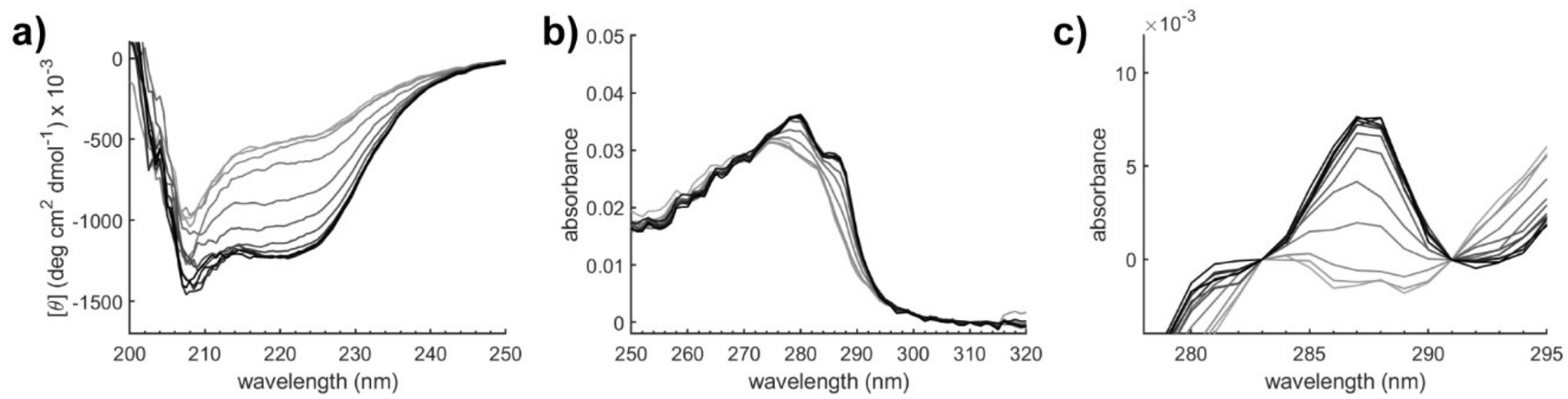

Figure S6. Spectra from titrations of wt LanM (20 μM) with Ca^{II}. Darker lines indicate more Ca^{II} added. Spectra were acquired by: **a)** titration with Ca^{II} measured by circular dichroism spectroscopy; **b)** titration with Ca^{II} measured by UV-visible spectroscopy. **c)** Spectra in **(b)** corrected by subtraction of a linear baseline between the absorbances at 283 and 291 nm. Spectra shown are from representative titrations; each stoichiometric determination was an average of three replicate experiments.

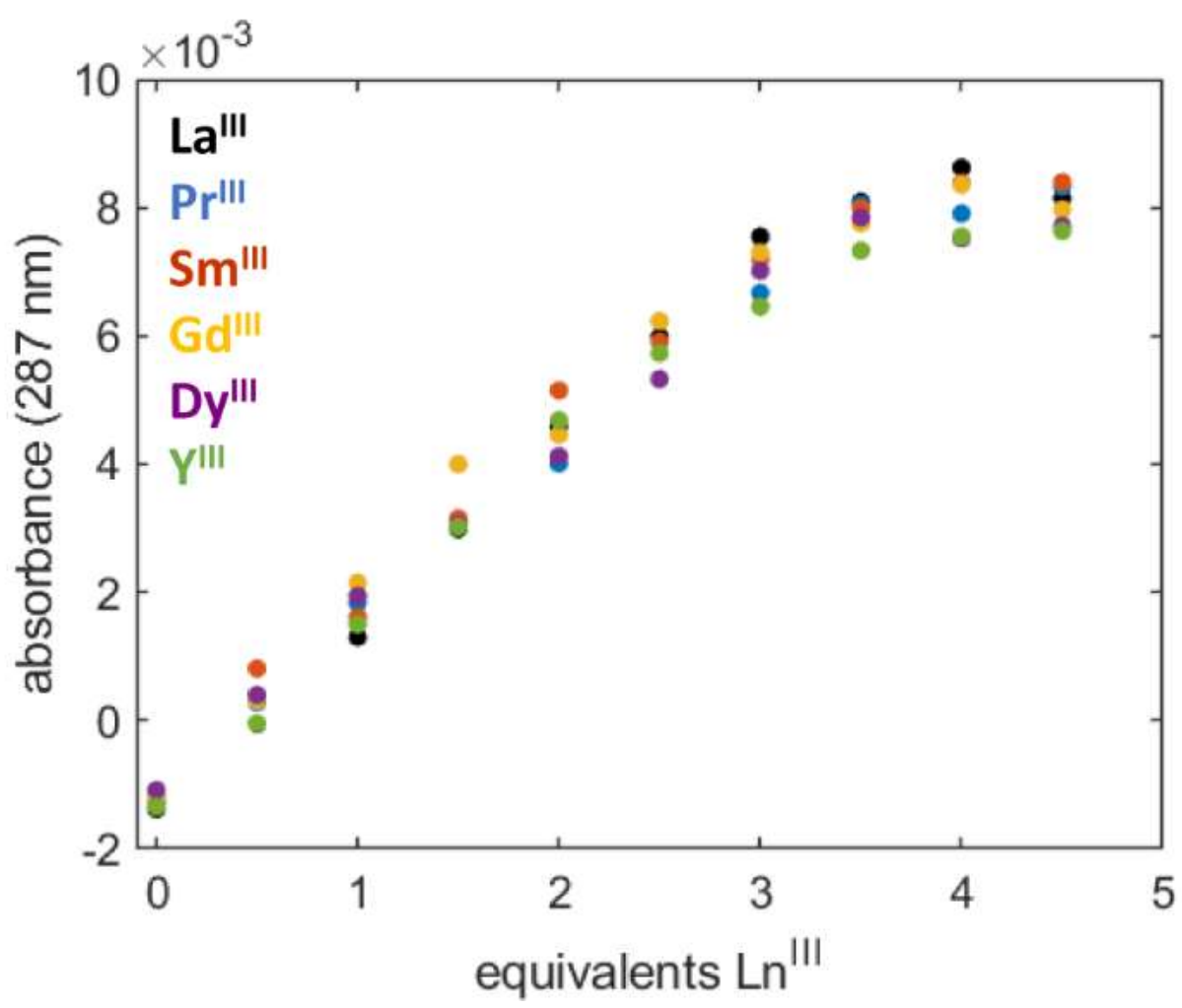

Figure S7. UV absorbance spectroscopic response of Y96 to stoichiometric binding of Ln^{III} ions to wt LanM (20 μM) at pH 7.0. Values reported result from subtraction of a linear baseline calculated between 283 and 291 nm, isolating the absorbance related to hydrogen bonding of Y96 from the underlying tyrosine absorbance. Each ion studied results in a similar spectroscopic change, suggesting that binding of each promotes the same protein tertiary structure change. Data shown are results of a single titration for each ion.

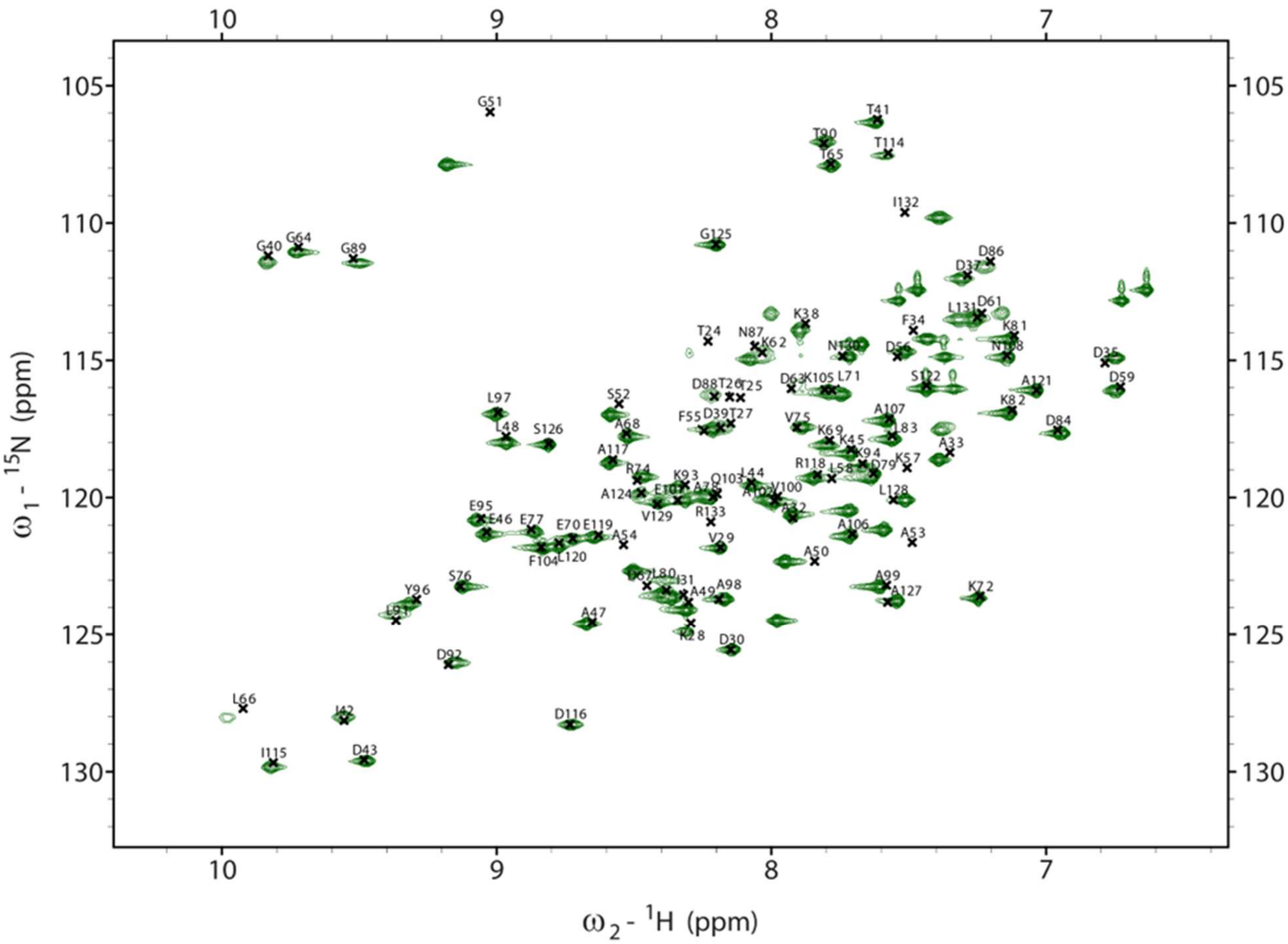

Figure S8. ^{1}H, ^{15}N HSQC NMR spectrum of wt LanM at pH 7.0 loaded with 3 equiv. Y^{III} (green). Labels indicate the positions of resonances from the previously published structure (Y^{III}-bound, C-terminally His_6-tagged LanM) under these conditions.[6] The small deviations for A50-A53 and I132 likely reflect the fact that these residues are at the junction of core helix α1 and the C-terminal helix, a region that was plausibly slightly affected by the presence of the His_6 tag in the prior study.

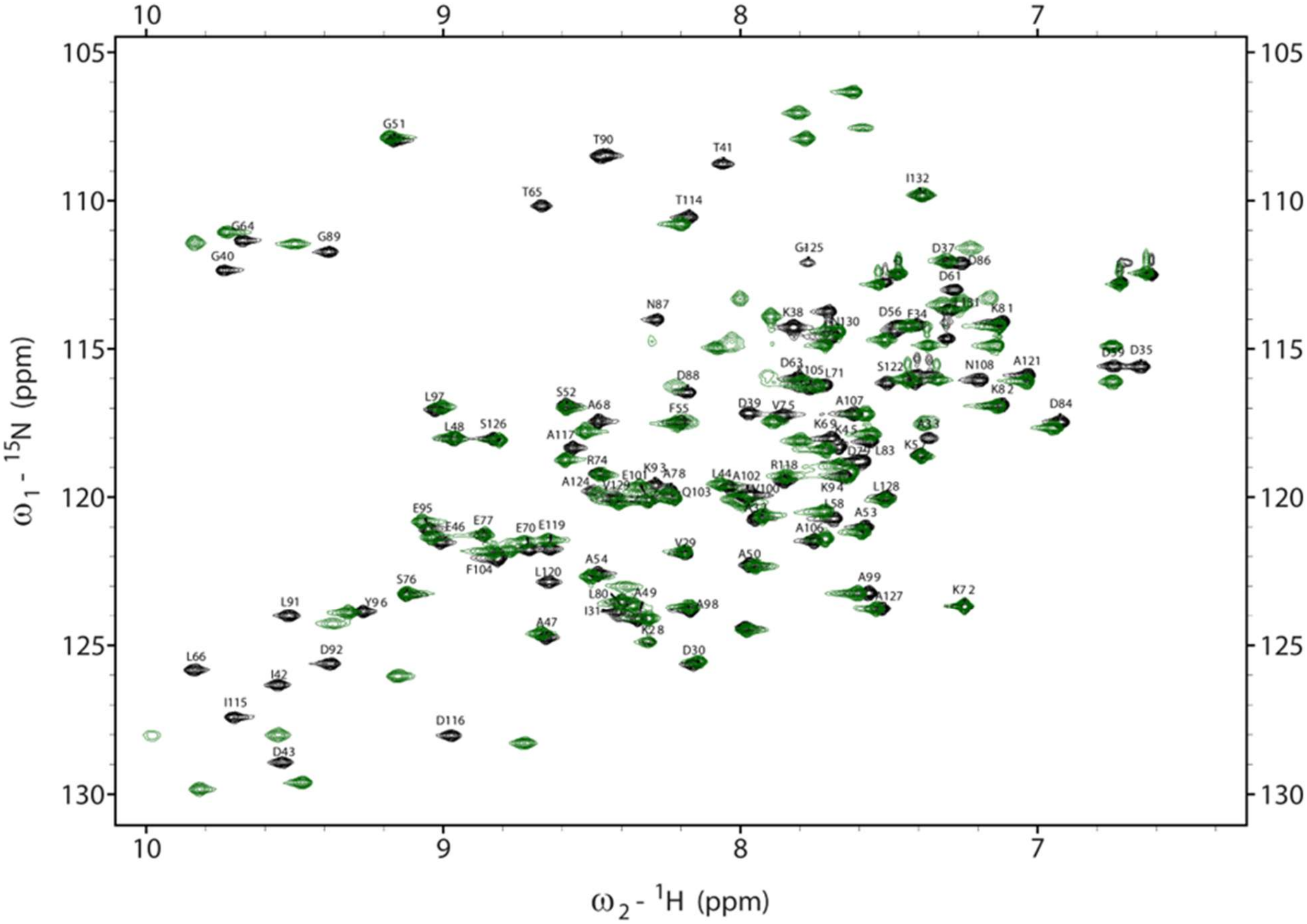

Figure S9. Overlaid ^{1}H, ^{15}N HSQC NMR spectra of wt LanM at pH 7.0 loaded with 3 equiv. La^{III} (black) and with 3 equiv. Y^{III} (green). The G_6, T_7, $(I/L)_8$ and D_9 residues of all four EF hands are among the most shifted in comparing the La^{III} and Y^{III} complexes.

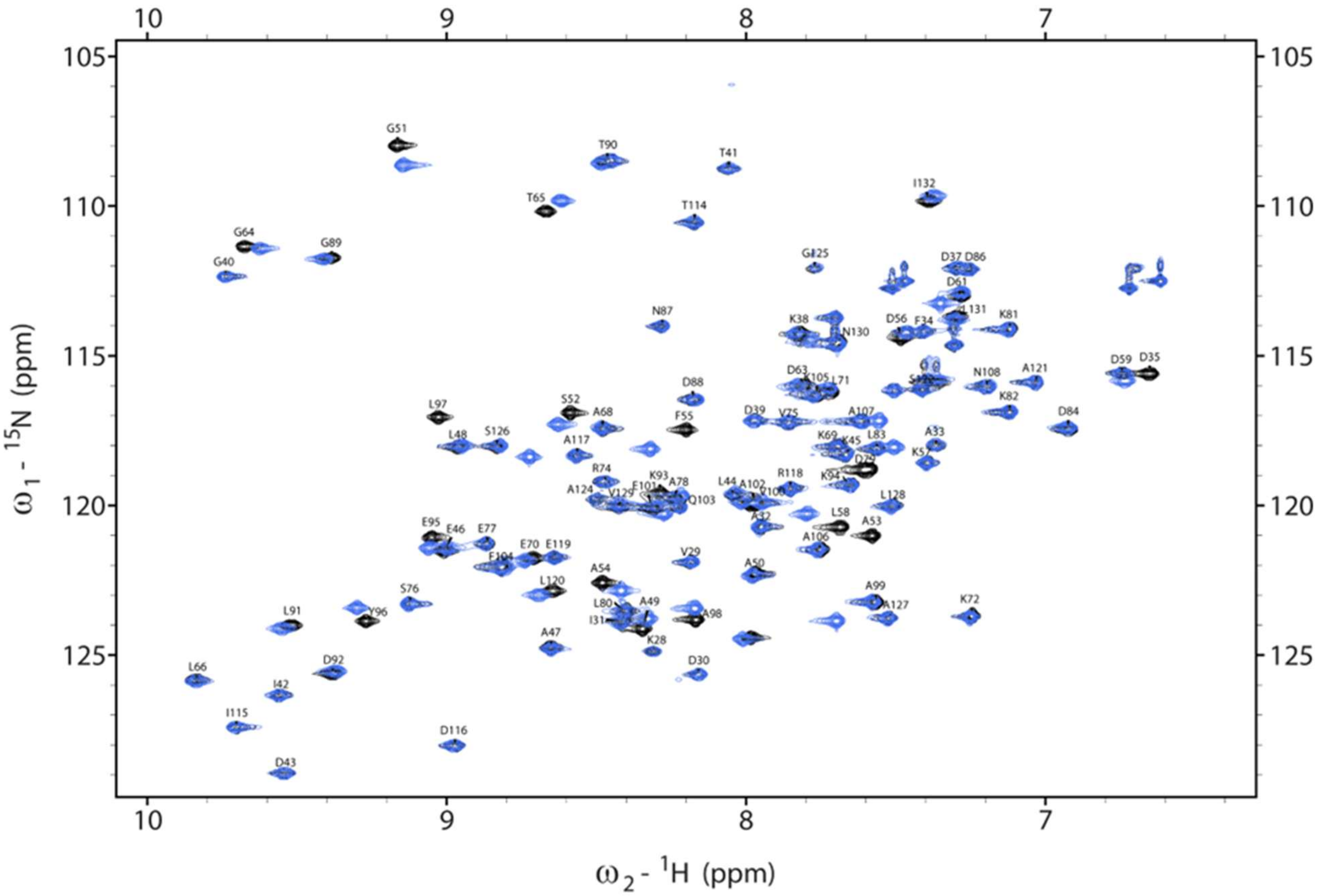

Figure S10. Overlaid ^{1}H, ^{15}N HSQC NMR spectra of wt (3 equiv. La^{III}, black) and K93A (2 equiv. La^{III}, blue) LanM at pH 7.0. Residues near the salt bridge involving K93 (in wt) in the vicinity of where core helix α2 (following EF3) packs with core helix α1 (between EF1 and EF2) are among the most shifted in comparing these proteins (see **Figure 5a**).

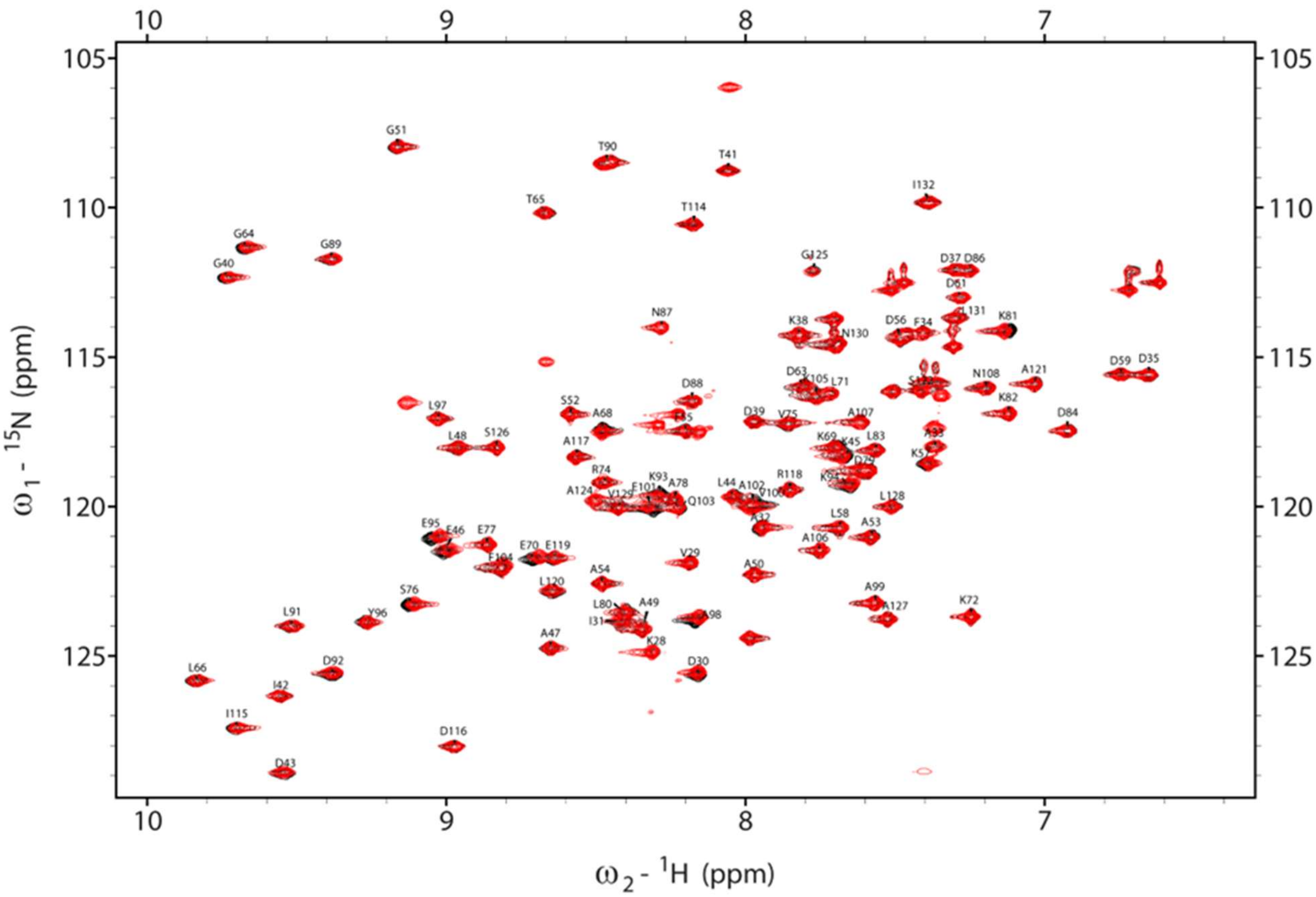

Figure S11. Overlaid ^{1}H, ^{15}N HSQC NMR spectra of wt LanM at pH 7.0 (3 equiv. La^{III}, black) and at pH 5.0 (2 equiv. La^{III}, red). See **Figure 5b** for a representation of the chemical shift perturbations.

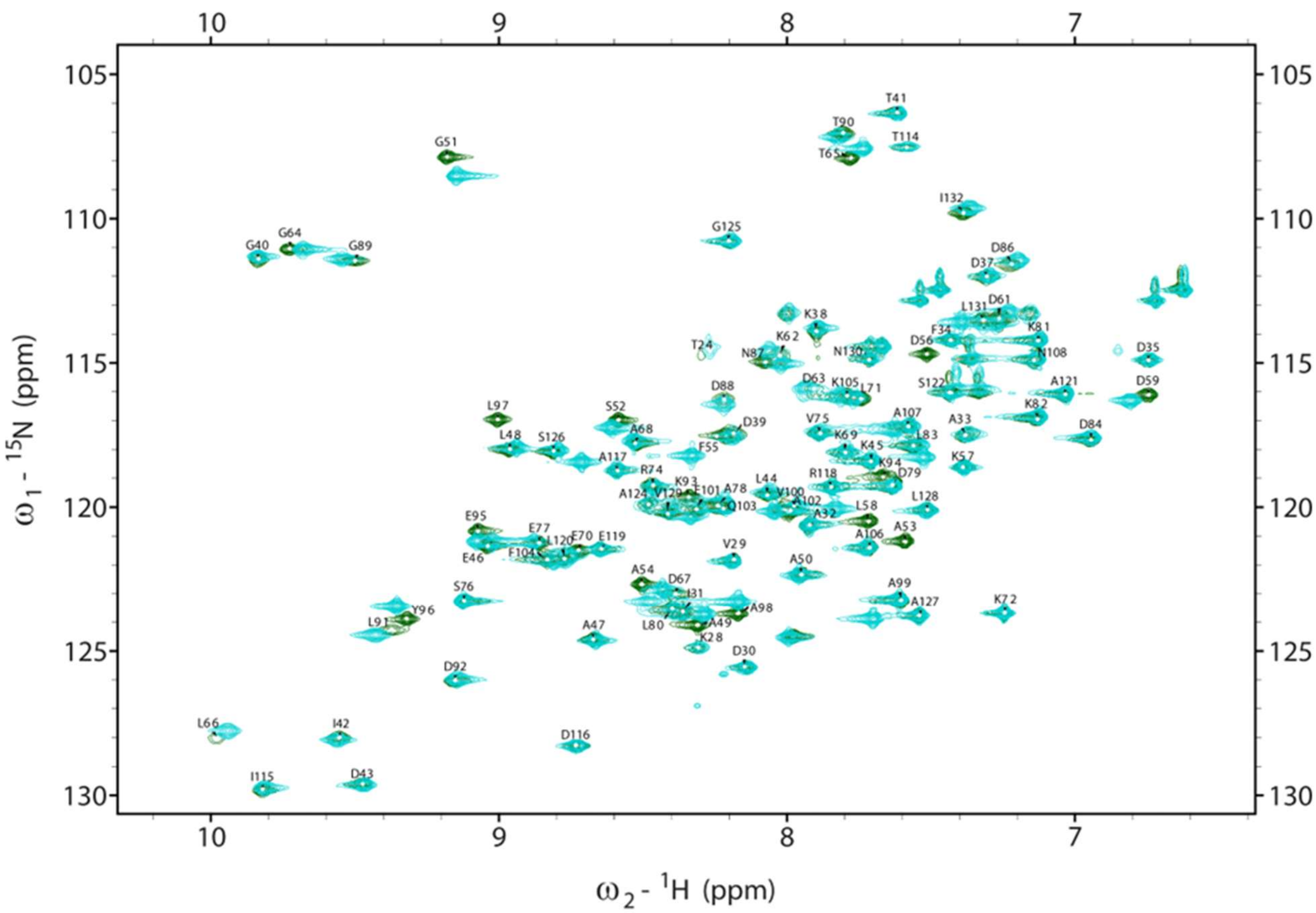

Figure S12. Overlaid ^{1}H, ^{15}N HSQC NMR spectra of wt (3 equiv. Y^{III}, green) and K93A (2 equiv. Y^{III}, teal) LanM at pH 7.0.

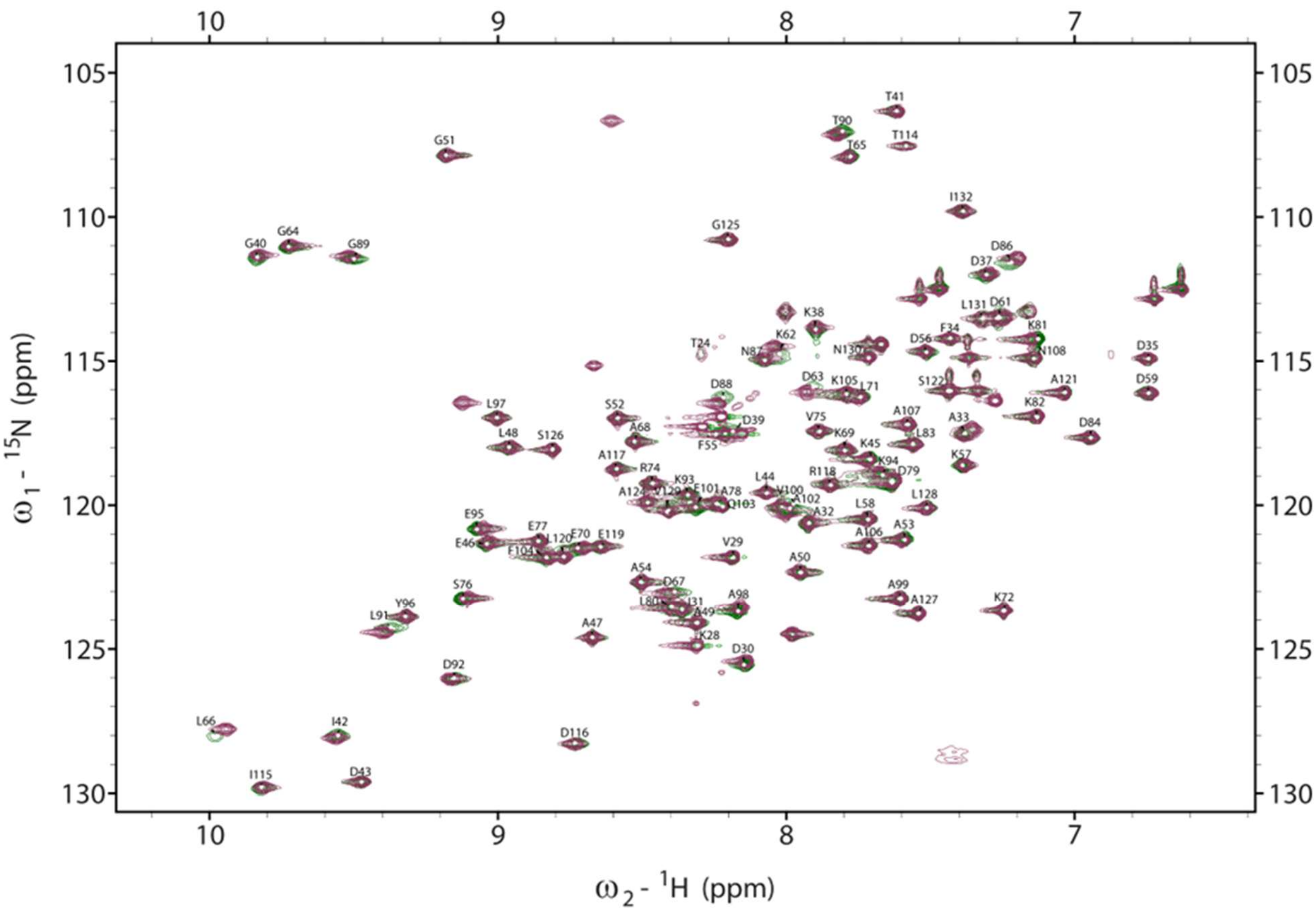

Figure S13. Overlaid ^{1}H, ^{15}N HSQC NMR spectra of wt LanM at pH 7.0 (3 equiv. Y^{III}, green) and at pH 5.0 (2 equiv. Y^{III}, plum).

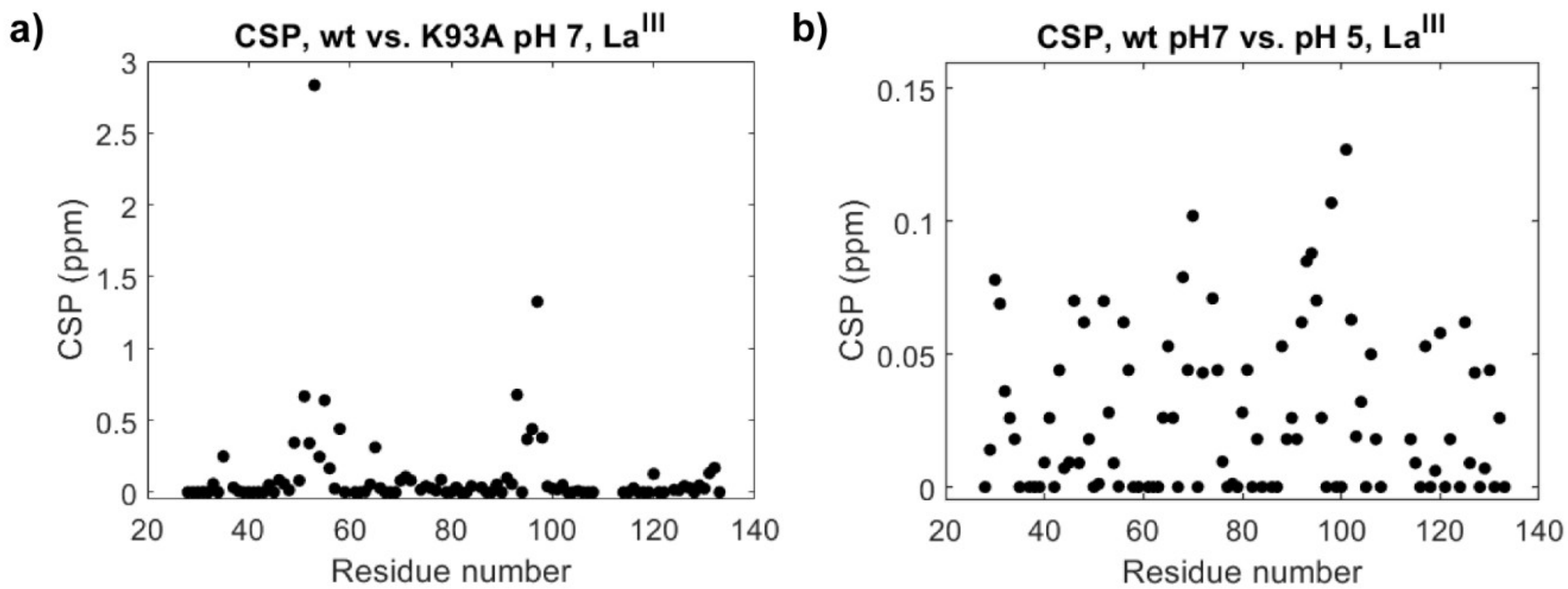

Figure S14. Backbone chemical shift perturbation (CSP) values for each residue of LanM, plotted by residue number for **a)** wt LanM with 3 equiv. La^{III} vs. K93A LanM with 2 equiv. La^{III} at pH 7.0 and **b)** wt LanM with 3 equiv. La^{III} at pH 7.0 vs. wt LanM with 2 equiv. La^{III} at pH 5.0. CSP was determined by the formula $\Delta\delta = [(\Delta\delta H)^2+0.2(\Delta\delta N)^2]^{1/2}$.

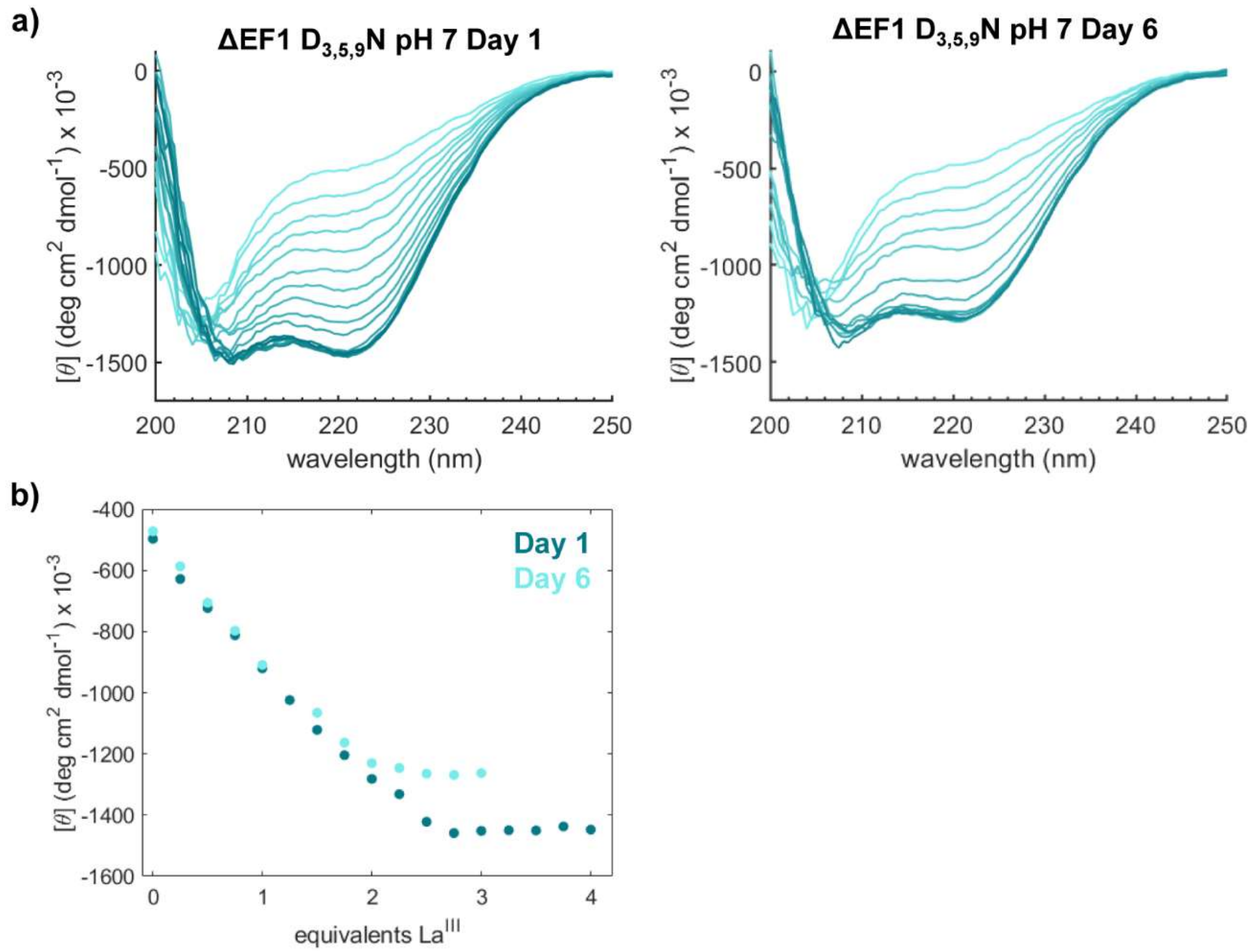

Figure S15. Impact of storage time on secondary structure formation in ΔEF1 $D_{3,5,9}N$ LanM. **a)** CD spectra acquired on day 1 and day 6 of incubation at 4 °C. Darker traces indicate higher La^{III} concentration. **b)** Signal at 222 nm indicating α-helix formation as a function of La^{III} added. Data shown are results of a single titration in each case.

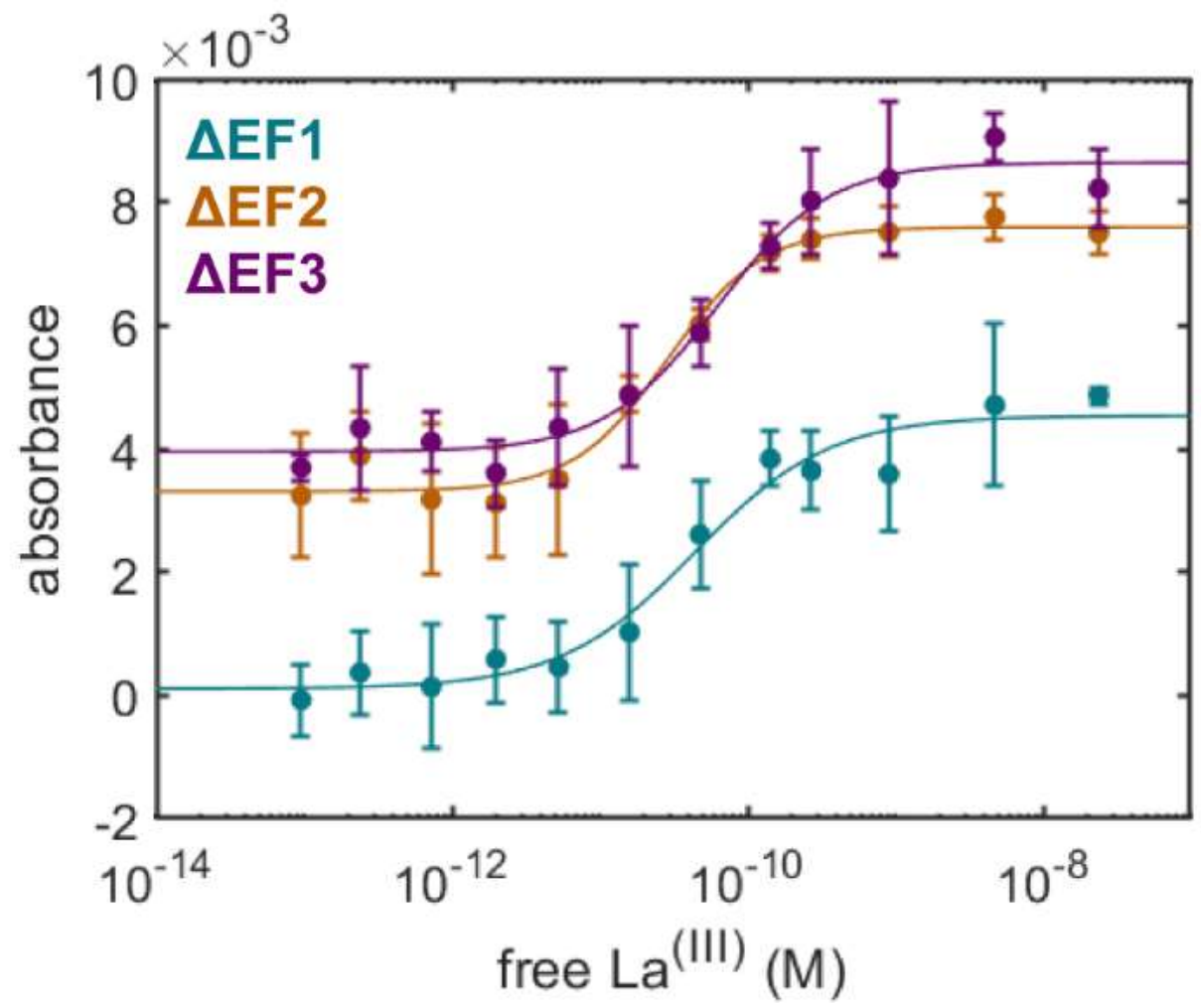

Figure S16. UV absorbance response of Y96 in $D_{3,5,9}N$ LanM variants (15 μM) to binding La^{III}, monitored at 287 nm, to determine K_D at pH 5.0. Solid lines represent fits of the data to the Hill equation. Error bars indicate the standard deviation from triplicate measurements of separate samples.

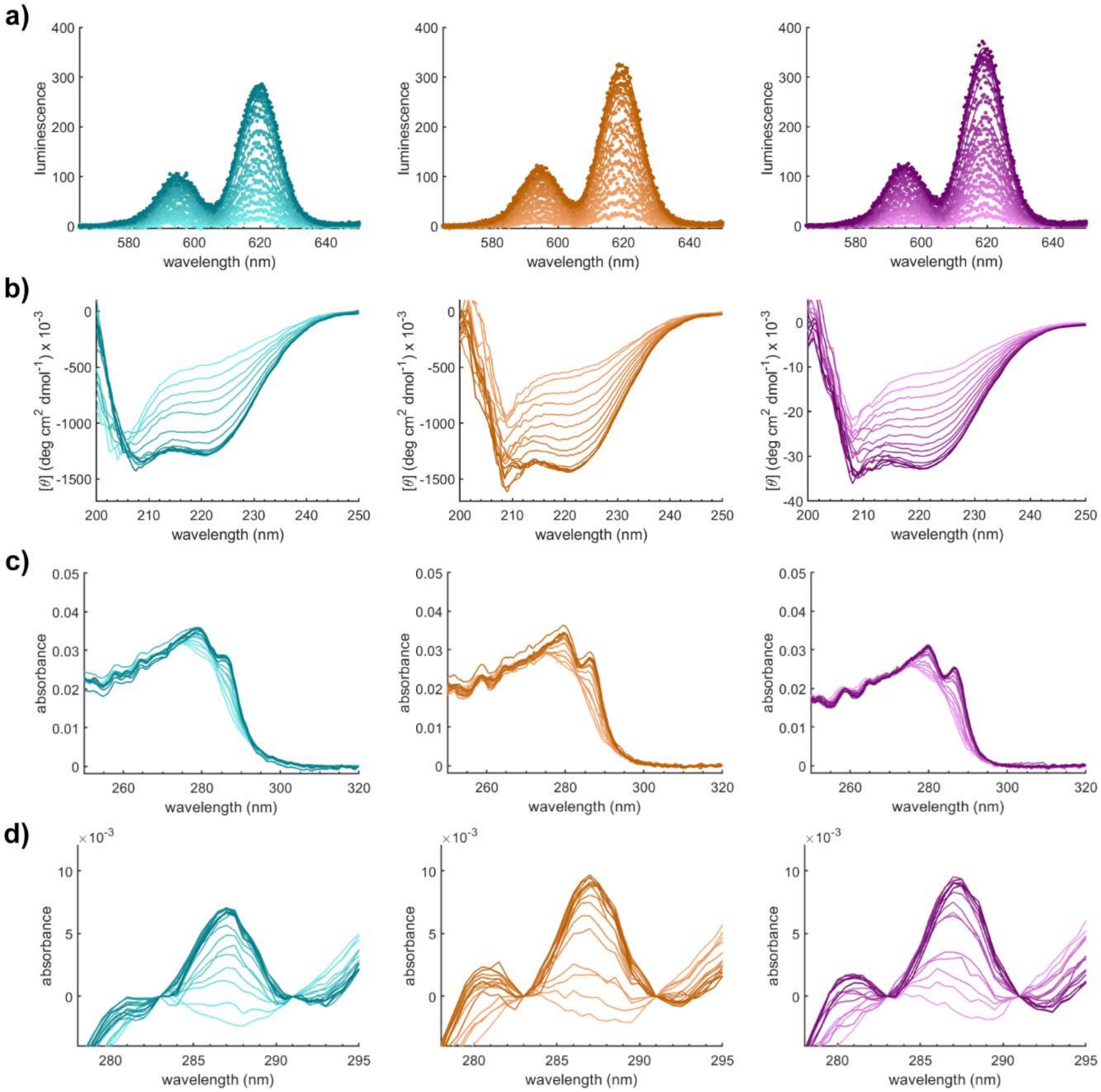

Figure S17. Spectra from stoichiometric titrations of LanM $D_{3,5,9}N$ variants at pH 7.0 (20 μM; left, ΔEF1; center, ΔEF2; right, ΔEF3). Darker lines indicate more Ln^{III} added. Spectra were acquired by: **a)** titration with Eu^{III} measured by luminescence spectroscopy with excitation at 394 nm; **b)** titration with La^{III} measured by circular dichroism spectroscopy; **c)** titration with La^{III} measured by UV-visible spectroscopy. **d)** shows the spectra in **c)** corrected by subtraction of a linear baseline between the absorbances at 283 and 291 nm. Spectra shown are from representative titrations; each stoichiometric determination was an average of three replicate experiments.

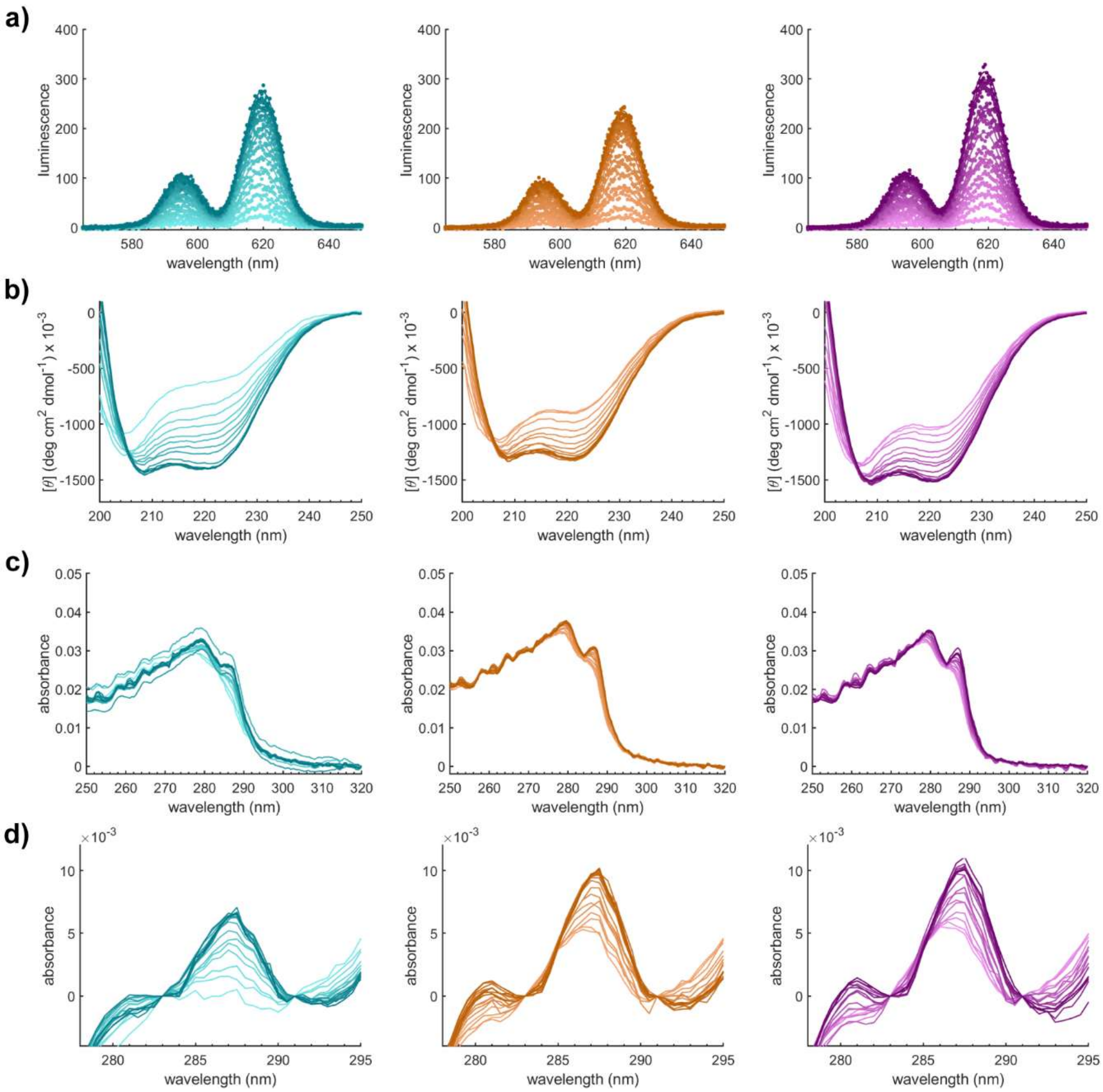

Figure S18. Spectra from stoichiometric titrations of LanM $D_{3,5,9}N$ variants at pH 5.0 (20 μM; left, ΔEF1; center, ΔEF2; right, ΔEF3). Darker lines indicate more Ln^{III} added. Spectra were acquired by: **a)** titration with Eu^{III} measured by luminescence spectroscopy with excitation at 394 nm; **b)** titration with La^{III} measured by circular dichroism spectroscopy; **c)** titration with La^{III} measured by UV-visible spectroscopy. **d)** shows the spectra in **c)** corrected by subtraction of a linear baseline between the absorbances at 283 and 291 nm. Spectra shown are from representative titrations; each stoichiometric determination was an average of three replicate experiments.

SUPPLEMENTARY REFERENCES

(1) Cotruvo, J. A., Jr.; Featherston, E. R.; Mattocks, J. A.; Ho, J. V.; Laremore, T. N. Lanmodulin: A highly selective lanthanide-binding protein from a lanthanide-utilizing bacterium. *J. Am. Chem. Soc.* **2018**, *140* (44), 15056-15061.
(2) Featherston, E. R.; Mattocks, J. A.; Tirsch, J. L.; Cotruvo, J. A., Jr. Heterologous expression, purification, and characterization of proteins in the lanthanome. *Methods Enzymol.* **2021**, *650*, 119-157.
(3) Mattocks, J. A.; Tirsch, J. L.; Cotruvo, J. A., Jr. Determination of affinities of lanthanide-binding proteins using chelator-buffered titrations. *Methods Enzymol.* **2021**, *651*, 23-61.
(4) Martell, A. E.; Smith, R. M. *Critical Stability Constants*; Plenum Press, 1974.
(5) Lee, W.; Tonelli, M.; Markley, J. L. NMRFAM-SPARKY: enhanced software for biomolecular NMR spectroscopy. *Bioinformatics* **2015**, *31* (8), 1325-1327.
(6) Cook, E. C.; Featherston, E. R.; Showalter, S. A.; Cotruvo, J. A., Jr. Structural basis for rare earth element recognition by *Methylobacterium extorquens* Lanmodulin. *Biochemistry* **2019**, *58* (2), 120-125.
